# Influence of the Initial Neutrophils to Lymphocytes and Platelets Ratio on the Incidence and Severity of Sepsis-Associated Acute Kidney Injury: A Double Robust Estimation Based on a Large Public Database

**DOI:** 10.3389/fimmu.2022.925494

**Published:** 2022-07-12

**Authors:** Wenyan Xiao, Zongqing Lu, Yu Liu, Tianfeng Hua, Jin Zhang, Juanjuan Hu, Hui Li, Yaohua Xu, Min Yang

**Affiliations:** ^1^ The 2nd Department of Intensive Care Unit, the Second Affiliated Hospital of Anhui Medical University, Hefei, China; ^2^ The Laboratory of Cardiopulmonary Resuscitation and Critical Care Medicine, The Second Affiliated Hospital of Anhui Medical University, Hefei, China; ^3^ Key Laboratory of Intelligent Computing and Signal Processing, Anhui University, Ministry of Education, Hefei, China; ^4^ School of Integrated Circuits, Anhui University, Hefei, China

**Keywords:** sepsis, neutrophils to lymphocytes and platelets ratio, acute kidney injury, competing risk model, double robust estimation

## Abstract

**Background:**

Acute kidney injury (AKI) is a frequent consequence of sepsis and has been linked to poor prognosis. In critically ill patients, the ratio of neutrophils to lymphocytes and platelets (N/LP) has been confirmed as an inflammation-related marker connected with the development of renal dysfunction. However, the effect of the N/LP ratio on the initiation and development of AKI in patients with sepsis remained unclear. The purpose of this study was to determine if the N/LP ratio on intensive care unit (ICU) admission was associated with the occurrence of sepsis-associated AKI (S-AKI) and severe AKI.

**Methods:**

Adult septic patients from the Medical Information Mart for Intensive Care-IV database were screened and classified into three categories (low, middle, or high) based on their N/LP ratio quartiles. The Cox proportional hazard and competing risk models were used to determine the risk of S-AKI in various N/LP groups, whilst the logistic regression model and restricted cubic splines (RCS) analysis were employed to investigate the link between N/LP ratios and the occurrence of severe AKI. Finally, we did a doubly robust estimation, a subgroup analysis, and a sensitivity analysis to determine the findings’ robustness.

**Results:**

We categorized 485, 968, and 485 septic patients into three groups based on their N/LP ratios: low, intermediate, and high. According the Cox proportional hazard model, the hazard rate (95% CI) for those in the middle and high N/LP groups on the incidence of S-AKI were 1.30(1.07, 1.58) and 1.27(1.02, 1.59), respectively, as compared to those in the low N/LP group. And the Fine-Gray proportional subdistribution hazards model indicated that mortality was not a substantial competing risk for S-AKI. Additionally, multivariate logistic regression revealed that the risk of severe AKI increased 1.83 fold in the high group compared to the low group. The RCS result also suggested that the probability of severe AKI rose significantly when N/LP > 9.5. The consistency of these findings was confirmed using doubly robust estimation. However, subgroup and sensitivity analyses revealed that the association between N/LP and the incidence of S-AKI, severe AKI varied considerably between different populations and diagnostic criteria.

**Conclusion:**

A raised initial N/LP level may induce the development of S-AKI and severe AKI within 7 days after ICU admission in septic patients. These influences were enhanced in elder, male, septic shock, and those with poor health condition. Furthermore, high NLP was more strongly connected to the risk of S-AKI and severe AKI in sepsis patients on the urine output-based AKI criteria than on the serum creatinine-based criteria.

## Introduction

According to the latest Sepsis 3.0 criteria (1), sepsis is defined as multiple organ dysfunction caused by an uncontrolled immune response to infection. Despite significant research efforts, sepsis continues to be one of the most challenging tasks for the healthcare system, with high morbidity and death globally. Based on evidence from previous epidemiological research, the worldwide incidence of sepsis is around 270 per 100,000 person-years (2). And the in-hospital mortality ranges from 17% to 26% (2), depending on the severity of the condition. As a common complication in sepsis patients, acute kidney damage (AKI) is usually results from tissue hypoperfusion, immune-inflammatory response dysregulation, and others (3, 4), with a incidence as high as 50% (5) and a 6-8 fold increased mortality compared to those with sepsis alone (6). As a result, it is necessary to initiate the early diagnosis and risk stratification of AKI in sepsis patients, which would contribute to effective interventions and a favorable prognosis.

Serum creatinine concentrations (SCr) and urine output (UO) are critical for clinical diagnosis of sepsis-associated AKI (S-AKI). However, due to its unique peak period and enhanced tubular secretion under stress, SCr appears to be an insensitive marker of AKI (7). At the same time, the UO is also particularly susceptible to varying factors other than the kidney, such as the circulating blood volume, urethral obstruction, and the use of diuretics. Thus, neither SCr nor UO are ideal and reliable indicators for S-AKI. Furthermore, numerous emerging technologies and approaches, including as machine learning (8, 9) and a series of novel biomarkers (7, 10), have been employed for S-AKI prediction and diagnosis. Nevertheless, it is regrettable that their actual effectiveness still requires extensive external validation and preclinical investigation. As noted previously, systemic inflammatory responses are intimately connected to the onset and development of S-AKI. The neutrophils to lymphocytes and platelets ratio (N/LP) is a low-cost indicator that can be obtained simply through routine blood tests and has been frequently applied in reflecting the body’s inflammatory state. Its utility as a predictor of COVID-19 prognosis (11) and AKI occurrence following abdominal and cardiovascular surgery has already been demonstrated (12, 13). A recent retrospective study also demonstrated that higher N/LP ratios were significantly associated with an increased risk of in-hospital mortality among S-AKI patients (14). However, the relationship between the initial N/LP ratio and the development or severity of AKI in sepsis patients remained unclear. Besides, a large cohort research by Bianchi NA et al. revealed discrepancy between SCr and UO criteria for AKI diagnosis and prognosis (15). And the most appropriate diagnostic criteria that satisfied the clinical application of the N/LP are likewise undefined.

Accordingly, this study intended to determine whether elevated N/LP is causally linked with S-AKI risk and severity within seven days after ICU admission by using several statistical methods in a large cohort of adult sepsis patients. Additionally, we further explored the consistency of these associations on specific population subgroups and different diagnostic criteria for AKI to provide a basis for application scenarios.

## Methods

### Data Sources

The data for this study were obtained from the Medical Information Mart for Intensive Care IV (v1.0) (MIMIC-IV 1.0) database, which is a large, open-access database. The MIMIC-IV contained electronic data from 382, 278 patients admitted to the Beth Israel Deaconess Medical Center in Boston, Massachusetts, between 2008 and 2019 (16), including demographics, vital signs, laboratory results, imaging examinations, microbial culture results, medication and procedure records, survival information, and a data dictionary. This critical care database has been approved by the Massachusetts Institute of Technology’s Institutional Review Boards and released on March 16, 2021. Despite the fact that a substantial amount of research has been done based on MIMIC-IV, it was necessary to obtain authorization prior to the using. We have completed the Collaborative Institution Training Initiative Program Course offered by the National Institutes of Health in the United States and obtained certification (Record ID: 38455175, 39691989). Since the MIMIC database is a publicly available anonymized database, approval for the ethical committee was exempted.

### Population Selection Criteria

Patients who met the criteria for sepsis at the time of ICU admission were eligible for enrollment. Sepsis and septic shock were defined according to the Third International Consensus Definitions for Sepsis and Septic Shock (Sepsis-3) (1), which included patients with a confirmed or suspected infection and a total SOFA score of 2 points. Simultaneously, suspected infection was defined as cases in which empiric antibiotic therapy was administered prior to or within three days of culture collection. Only data from the patient’s first admission was used if they had multiple admission records. Minors (those under the age of 18) and those who were discharged or died within 24 hours of ICU admission were excluded. To avoid bias, patients with the following conditions were also excluded: (1) those with a blood system disease, such as aplastic anemia or various acute leukemia; (2) those with major immune diseases, including lymphoma, acquired immune deficiency syndrome, solid metastatic tumor, malignant tumor, and systemic lupus erythematosus; (3) those with pre-existing end-stage renal disease; (4) those with cirrhosis-induced hypersplenism; (5) those who received an acute renal damage diagnosis within the first 24 hours of ICU admission. When the patient was discharged, all associated comorbidities were identified using the International Classification of Diseases, Ninth Revision (ICD-9) and Tenth Revision (ICD-10) diagnosis codes.

### Data Collection and Classification

Clinical data for the included patients were extracted from MIMIC IV using PostgreSQL programming (v4.21). Following that, STATA software (v15.1) was used to integrate, process, and classify the data based on the particular hadm id or stay id code. These clinical data included demographic information, associated comorbidities, infection locations, illness severity score, laboratory test variables, therapies administered, and endpoints, as following: age, gender, ethnic origin, type of care unit, presence of myocardial infarction, congestive heart failure, cirrhosis combined with non-hypersplenism, chronic obstructive pulmonary disease, or diabetes, presence of lower respiratory infection, genitourinary tract infection, intra-abdominal infection, bacteremia, skin and soft tissue infection, musculoskeletal infection, biliary tract infection, fungal infection, platelet count, neutrophil absolute value, lymphocyte absolute value, white blood cell count (WBC), serum creatinine (SCr), blood urea nitrogen (BUN), glucose, serum potassium, sodium, chloride, serum anion gap (AG), serum bicarbonate, Simplify the Acute Physiological Scores II (SAPS II), the Sequential Organ Failure Assessment (SOFA) excluding the coagulation system, the Charlson comorbidity index, the duration of ICU stay, the use of vasoactive medication, continuous renal replacement therapy (CRRT), or invasive mechanical ventilation (MV) during the follow-up period, the length of ICU stay, the 7-day mortality, the 28-day mortality, all-cause ICU mortality, the incidence of AKI, and the AKI stage. Except for neutrophils, lymphocytes, and platelet count, the lowest values of the aforesaid laboratory markers were retrieved within 24 hours of ICU admission. Patients with any missing values were discarded; values falling below the 1st percentile or above the 99th percentile were deemed outliers and were deleted before analysis. The formula for calculating N/LP was previously described as [Neutrophil count(10^9^/L)*100]/[Lymphocyte count(10^9^/L)*Platelet count(10^9^/L)] (11). The mean of neutrophils, lymphocytes, and platelet count within the first 24 h after ICU admission were adopted in N/LP the computational process. Following that, all participants were classified into three categories based on their N/LP quartile range: low (N/LP=2.8718, 25th), middle (N/LP=10.4128, 25th-75th), and high (N/LP>10.4128, >75th).

### Outcomes

The study’s objectives were to determine the potential connection of different initial N/LP levels with the prevalence of S-AKI and severe AKI within 7 days after ICU admission in sepsis patients. Primary outcome was the S-AKI incidence; the secondary outcome was the risk of severe AKI. The duration of follow-up was defined as the period from ICU admission to the onset of AKI or death. While the overall time of follow-up for patients who survived without developing AKI was seven days. AKI was defined by the 2012 Kidney Disease: Improving Global Outcomes Clinical Practice Guidelines (KDIGO) as an elevated in serum creatinine of 0.3 mg/dL within 48 hours or a raise of at least 1.5 times the baseline level in the preceding seven days, or a decrease in UO to less than 0.5 ml/kg/h for more than 6 hours (17). If one or more of these criteria were met, AKI could be diagnosed; the highest stage among the SCr and UO criteria would be considered the final stage for those who met both criteria (15). And severe AKI was defined as stages 2 and 3 of AKI.

### Independent Variable and Covariates

The independent variable was the varying levels of N/LP. Any significant variables associated with the outcomes through univariate analyses as well as those variables that seemed clinically important were considered as modifying covariates for subsequent multivariate analysis. Finally, age, gender, initial SAPS II, SOFA scores excluding coagulation system, Charlson comorbidity index, serum AG, serum bicarbonate, glucose, serum potassium, serum sodium, serum chloride, BUN, SCr, and usage of vasoactive medicine, CRRT, and invasive-MV were considered as confounders by statistical analyses and clinical judgment.

### Statistical Analysis

We performed a normality test (*Agostino tests*), followed by a descriptive data analysis. Continuous variables were expressed as mean (standard deviation), while nonparametric variables were expressed as the median (interquartile ranges, IQR) and were compared using the one-way *ANOVA test* or nonparametric *Kruskal-Wallis test*. Categorical variables are expressed as a frequency (percentage) and were compared using the*X^2^* or *Rank-Sum test*.

We used the Kaplan-Meier (K-M) method to calculate the cumulative incidence rates and corresponding 95% confidence intervals (CIs) for each N/LP group and then plotted the cumulative incidence curves with the log-rank test for significance. The influence of the initial N/LP on AKI risk was then investigated using three Cox proportional hazard models with varied degrees of covariate adjustment. Firstly, a univariate analysis was conducted in Model 1 without adjusting for any covariates. Secondly, Model 2 modified with all sixteen factors discussed previously. Thirdly, in order to select the ideal model, we used a stepwise backward approach in multivariate analysis based on the Akaike information criteria and evaluated the possibility of collinearity in Model 3 by variance inflation factors (VIF). Additionally, variables with a VIF greater than 5 were excluded (15). Considering the death as a competing risk for the S-AKI occurrence, the cumulative incidence functions according to the Fine-Gray test was utilized to calculate the cumulative incidence for each of three groups in competing risk models and in turn, assess the stability of Cox proportional hazard model results (18). Similarly, we drew the cumulative incidence curve using the R package ‘cmprsk’ and then conducted Gray’s test to compare the AKI risk between the N/LP groups.

Logistic regression model was implied to assess the influence of different initial N/LP levels on the severity of AKI. Additionally, given the inevitable information loss and change of dose-response relationship when artificially stratifying continuous variables, we modeled the potential nonlinear effect of N/LP at the continuity level using restricted cubic splines analysis (RCS) (19). We used RCS with five knot corresponding to 5th, 35th, 50th, 65th, and 95th percentile after adjusting all 16 covariates. The reference point was set at the 2.8718 (25th).

To further validate the aforementioned findings, double robust estimation models were performed. Sixteen covariates were included in the propensity scoring model to obtain the propensity scores using logistic regression. We then re-weighted the observations across the N/LP groups using inverse probability treatment weighting (IPTW) to create three groups that were similar for all covariates (20). The standardized mean difference of effect sizes (SMDs) were calculated to reflect the differences between the original and the IPTW cohorts, and an SMD with an absolute value greater than 0.1 after propensity scores weighting could be considered evidence of imbalance. Weighted regression on all the confounders included in the propensity scoring model was conducted, thereby obtaining double robust estimators in different N/LP groups.

Subgroup analyses according to age (<65, or ≥65 years), gender (male, or female), septic shock (yes, or no), SOFA scores (<5, or ≥5), SAPS II (<35, or ≥35), and Charlson comorbidity index (<5, or ≥5) were conducted respectively to examine the strata effect and potential interactions. Finally, considering the significant inconsistency between SCr and urine output criteria in AKI diagnosing and grading, sensitivity analysis was performed to re-evaluate the association of the initial N/LP Levels with the AKI and severe AKI occurrence in individual AKI criteria (SCr or UO criteria). All statistical analyses were performed using STATA 15.1 (College Station, Texas) and R 3.6.2 (Chicago, Illinois) software. The primary R package used in this study included *survival, survminer, cmprsk, tableone, foreign, ipw, twang, MatchIt, Hmisc, rms, glmnet, MASS, VIM, ggplot2*. The *p* values with < 0.05 were taken as statistically significant (two-sided).

## Results

### Characteristics of Included Sepsis Participants

**Figure 1:**
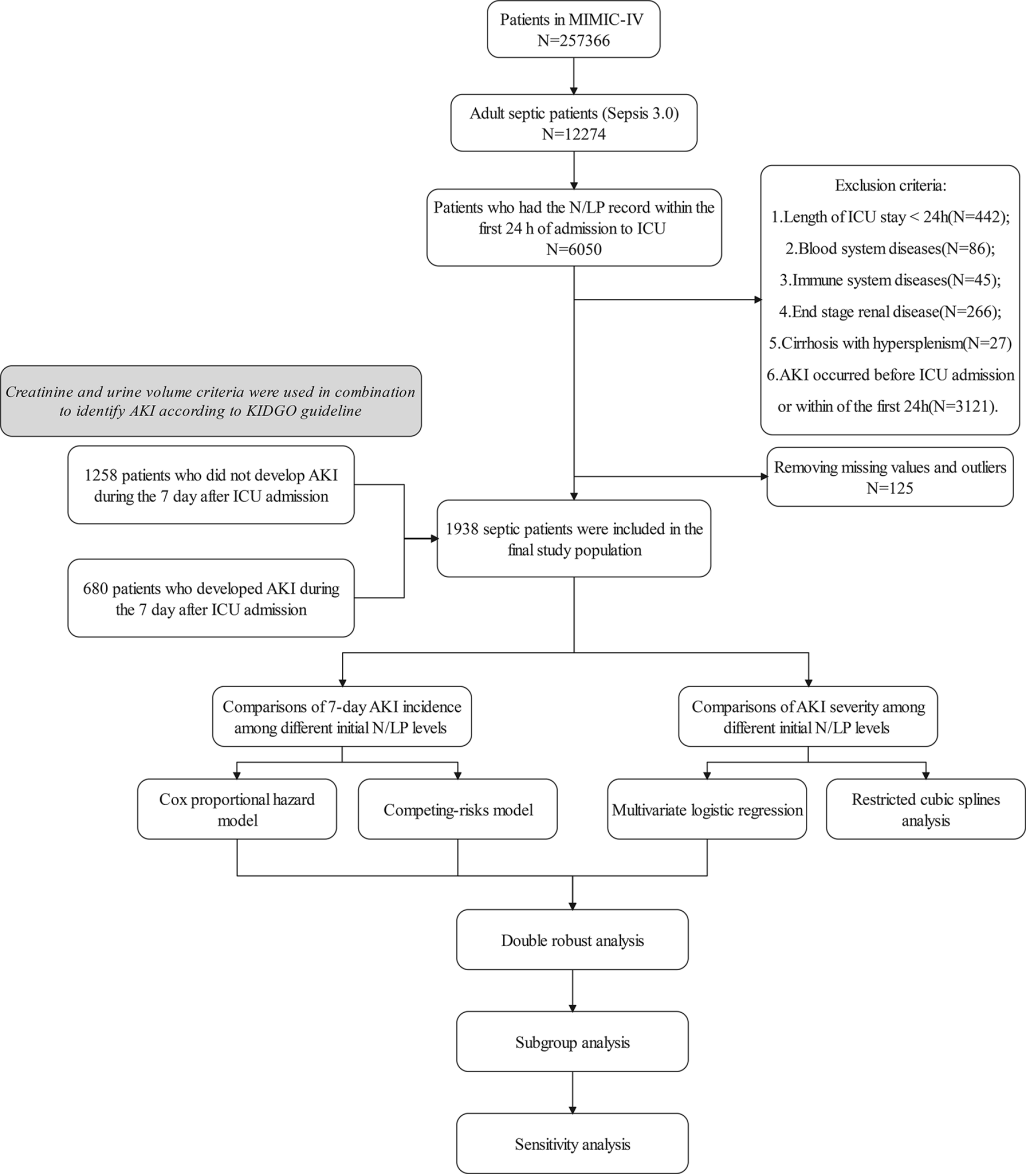
Flowchart for analysis of the current study.

With in MIMIC-IV database, a total of 12274 patients met the Sepsis 3.0 criteria; and finally, 1938 patients were included in this study (**Figure 1**). The 25% quantile, median, and 75% quantile of the N/LP were given by 2.8718, 5.2734, and 10.4128 respectively. Of those enrolled, 485 patients were assigned to the low N/LP group, 968 to the middle N/LP group, and 485 to the high N/LP group using predefined grouping criteria. The baseline characteristics of participants were given in **Table 1** according to respective N/LP groups. We found that the high N/LP group had a greater proportion of female (*P*=0.018), Caucasian (*P*=0.002), cirrhosis without hypersplenism (*P*=0.002) compared to other groups. With regard to the source of infection, the principal sites of the infection in high N/LP group were lower respiratory (P<0.001), genitourinary tract (*P*=0.009), intra-abdominal (*P*=0.001), skin and skin structure infection (*P*=0.017). Furthermore, the length of ICU stay (*P*=0.001) and 28-day mortality (*P*=0.001) increased significantly in high N/LP group.

**Table 1:** Baseline demographic and clinical characteristics by different N/LP level in sepsis patients.

Variables	Overall	Low group (N/LP<=2.8718)	Middle group (2.8718<N/LP<10.4128)	High group (N/LP>=10.4128)	*P*
**N**	1938	485	968	485	
**Age (year)**	65.99 [54.48, 78.37]	66.44 [53.29, 77.98]	65.96 [54.78, 78.45]	65.34 [54.71, 78.40]	0.688
**Gender (%)**					0.018
Female	1054 (54.4)	241 (49.7)	528 (54.5)	285 (58.8)	
Male	884 (45.6)	244 (50.3)	440 (45.5)	200 (41.2)	
**Ethnicity (%)**					0.002
AMERICAN INDIAN/ALASKA NATIVE	6 (0.3)	3 (0.6)	1 (0.1)	2 (0.4)	
ASIAN	75 (3.9)	18 (3.7)	32 (3.3)	25 (5.2)	
BLACK/AFRICAN AMERICAN	160 (8.3)	57 (11.8)	75 (7.7)	28 (5.8)	
HISPANIC/LATINO	71 (3.7)	25 (5.2)	27 (2.8)	19 (3.9)	
WHITE	1290 (66.6)	302 (62.3)	649 (67.0)	339 (69.9)	
UNKNOWN	230 (11.9)	50 (10.3)	134 (13.8)	46 (9.5)	
OTHER	106 (5.5)	30 (6.2)	50 (5.2)	26 (5.4)	
**First_careunit (%)**					<0.001
CVICU	267 (13.8)	89 (18.4)	166 (17.1)	12 (2.5)	
CCU	107 (5.5)	34 (7.0)	55 (5.7)	18 (3.7)	
MICU	617 (31.8)	137 (28.2)	292 (30.2)	188 (38.8)	
MICU/SICU	579 (29.9)	131 (27.0)	270 (27.9)	178 (36.7)	
Neuro Intermediate	35 (1.8)	14 (2.9)	10 (1.0)	11 (2.3)	
Neuro Stepdown	16 (0.8)	5 (1.0)	9 (0.9)	2 (0.4)	
Neuro SICU	28 (1.4)	8 (1.6)	17 (1.8)	3 (0.6)	
SICU	170 (8.8)	40 (8.2)	87 (9.0)	43 (8.9)	
Trauma SICU TSICU	119 (6.1)	27 (5.6)	62 (6.4)	30 (6.2)	
**Comorbidity (%)**					
Myocardial infarction	267 (13.8)	53 (10.9)	155 (16.0)	59 (12.2)	0.015
Congestive heart failure	438 (22.6)	106 (21.9)	221 (22.8)	111 (22.9)	0.902
Peripheral vascular disease	164 (8.5)	39 (8.0)	91 (9.4)	34 (7.0)	0.282
Cerebrovascular disease	220 (11.4)	74 (15.3)	94 (9.7)	52 (10.7)	0.006
COPD	478 (24.7)	130 (26.8)	230 (23.8)	118 (24.3)	0.438
Cirrhosis without hypersplenism	135 (7.0)	21 (4.3)	65 (6.7)	49 (10.1)	0.002
Diabetes	498 (25.7)	135 (27.8)	246 (25.4)	117 (24.1)	0.4
**Infection sites (%)**					
Lower respiratory infection	528 (27.2)	106 (21.9)	251 (25.9)	171 (35.3)	<0.001
Genitourinary tract infection	373 (19.2)	82 (16.9)	175 (18.1)	116 (23.9)	0.009
Intra abdominal infection	67 (3.5)	4 (0.8)	38 (3.9)	25 (5.2)	0.001
Bacteremia	65 (3.4)	19 (3.9)	28 (2.9)	18 (3.7)	0.522
Skin and skin structure infection	99 (5.1)	13 (2.7)	55 (5.7)	31 (6.4)	0.017
Musculoskeletal infection	18 (0.9)	6 (1.2)	5 (0.5)	7 (1.4)	0.158
Biliary tract infection	13 (0.7)	2 (0.4)	6 (0.6)	5 (1.0)	0.48
Systemic fungal infection	98 (5.1)	18 (3.7)	46 (4.8)	34 (7.0)	0.053
Other infection	923 (47.6)	271 (55.9)	489 (50.5)	163 (33.6)	<0.001
**Laboratory tests^a^**					
Platelet_mean (K/uL)	166.75 [119.50, 236.00]	215.00 [149.33, 287.00]	167.75 [123.92, 236.08]	128.50 [87.00, 183.50]	<0.001
Lymphocytes_mean (K/uL)	29.50 [1.21, 103.96]	69.03 [2.10, 154.28]	50.30 [1.26, 107.73]	2.80 [0.52, 44.66]	<0.001
Neutrophils_mean (K/uL)	288.47 [10.24, 990.07]	181.26 [7.27, 655.20]	415.75 [10.67, 1071.57]	200.00 [13.41, 1191.08]	<0.001
WBC_max (K/uL)	13.90 [9.60, 19.10]	11.40 [7.70, 15.50]	14.30 [10.30, 19.00]	16.10 [11.20, 23.00]	<0.001
Aniongap_max	16.00 [13.00, 19.00]	15.00 [13.00, 18.00]	16.00 [13.00, 19.00]	17.00 [14.00, 20.00]	<0.001
Bicarbonate_min (mEq/L)	21.00 [18.00, 23.00]	21.00 [19.00, 24.00]	21.00 [18.00, 23.00]	20.00 [17.00, 23.00]	<0.001
Bun_max (mg/dL)	21.00 [14.00, 36.00]	18.00 [13.00, 32.00]	20.50 [14.00, 34.00]	26.00 [17.00, 43.00]	<0.001
Chloride_max (mEq/L)	107.00 [103.00, 111.00]	107.00 [104.00, 110.00]	108.00 [103.00, 111.00]	107.00 [103.00, 111.00]	0.222
Creatinine_max (μmol/L)	1.10 [0.80, 1.60]	1.00 [0.70, 1.50]	1.10 [0.80, 1.50]	1.10 [0.80, 1.70]	<0.001
Glucose_max (mg/dl)	141.00 [115.00, 189.00]	135.00 [110.00, 176.00]	140.50 [115.00, 191.00]	149.00 [122.00, 200.00]	<0.001
Sodium_max (mEq/L)	140.00 [137.00, 143.00]	140.00 [137.00, 142.00]	140.00 [137.00, 142.00]	140.00 [137.00, 143.00]	0.866
Potassium_max (K/uL)	4.40 [4.00, 4.80]	4.40 [4.10, 4.90]	4.40 [4.00, 4.90]	4.30 [3.90, 4.70]	<0.001
**Severity scoring**					
SAPS II	35.00 [28.00, 43.00]	34.00 [26.00, 41.00]	35.00 [28.00, 42.25]	37.00 [30.00, 45.00]	<0.001
SOFA_exclude platelet	4.00 [3.00, 7.00]	4.00 [3.00, 6.00]	4.00 [3.00, 7.00]	5.00 [3.00, 7.00]	0.001
Charlson comorbidity index	5.00 [3.00, 7.00]	5.00 [3.00, 7.00]	5.00 [3.00, 7.00]	6.00 [4.00, 8.00]	0.028
**Treatments**					
Vasoactive drug (%)	864 (44.6)	201 (41.4)	447 (46.2)	216 (44.5)	0.231
Invasive ventilation (%)	666 (34.4)	159 (32.8)	354 (36.6)	153 (31.5)	0.115
CRRT (%)	7 (0.4)	1 (0.2)	3 (0.3)	3 (0.6)	0.525
**Endpoints**					
AKI (%)	680 (35.1)	141 (29.1)	360 (37.2)	179 (36.9)	0.006
AKI_stage (%)					0.004
Stage 1	260 (13.4)	62 (12.8)	145 (15.0)	53 (10.9)	
Stage 2	339 (17.5)	66 (13.6)	172 (17.8)	101 (20.8)	
Stage 3	81 (4.2)	13 (2.7)	43 (4.4)	25 (5.2)	
Length of ICU stay (day)	2.43 [1.63, 4.18]	2.23 [1.53, 3.67]	2.45 [1.63, 4.04]	2.73 [1.70, 5.14]	0.001
Mortality_ICU (%)	96 (5.0)	17 (3.5)	46 (4.8)	33 (6.8)	0.056
Mortality_ICU7 (%)	77 (4.0)	13 (2.7)	38 (3.9)	26 (5.4)	0.101
Mortality_ICU28 (%)	148 (7.6)	21 (4.3)	74 (7.6)	53 (10.9)	0.001

Categorical data were presented as frequency (percentage), parametric continuous data were presented as median (interquartile ranges), whereas non-parametric continuous data were presented as median (interquartile ranges);

CVICU, Cardiac Vascular Intensive Care Unit; CCU, Coronary Care Unit; MICU, Medical Intensive Care Unit; MICU/SICU, Medical/Surgical Intensive Care Unit; SICU, Surgical Intensive Care Unit; COPD, Chronic Obstructive Pulmoriary Disease; SOFA, Sequential Organ Failure Assessment; SAPS II, Simplified acute physiology score II; AKI, Acute kidney injury; CRRT, continuous renal replacement therapy; ICU, intensive care unit.

The characteristics of patients in whom S-AKI occurred and patients in whom S-AKI did not occur were summarized in **Supplementary Table 1**. Patients with AKI were elder, and had more comorbidities such as cardio-cerebrovascular disease and chronic obstructive pulmonary disease. Besides, a higher proportion of lower respiratory infection and organ dysfunction were also observed in those not developing AKI.

### The Incidence of S-AKI in Various N/LP Groups

**Figure 2:**
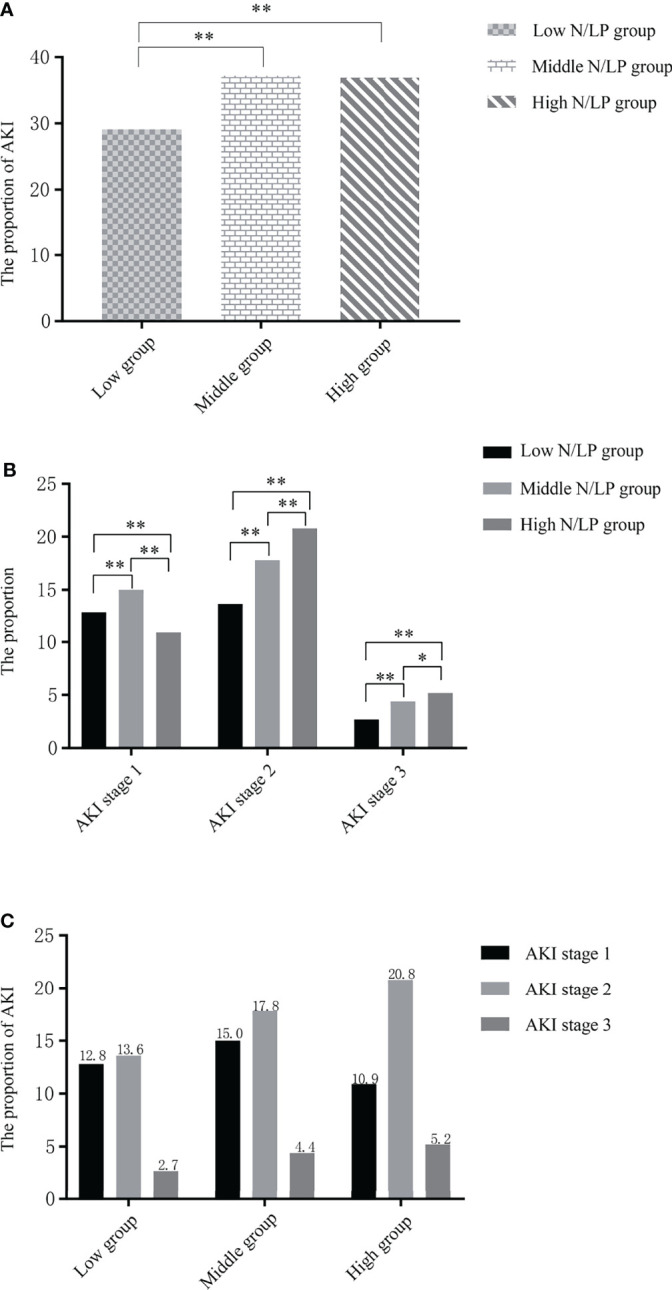
**(A)** The incidence of sepsis-associated acute kidney injury (S-AKI) in each N/LP group were 29.1% (low N/LP group), 37.2% (middle N/LP group) and 36.9% (high N/LP group), respectively (*P*=0.006); **(B)** the comparison in the proportion of each N/LP level in different AKI stages, the rates of stages 1, 2, 3 were 12.8%, 13.6, 2.7% in low N/LP group, and 15%, 17.8%, 4.4% in middle N/LP group, and 10.9%, 20.8%, 5.2%; **(C)** the proportion of each AKI stage in different N/LP groups. AKI and corresponding stage are diagnosed based on serum creatinine and urine output criteria. Statistical analysis by *Rank-Sum test;* *, *P*<0.05; **, *P*<0.001.

**Figure 3:**
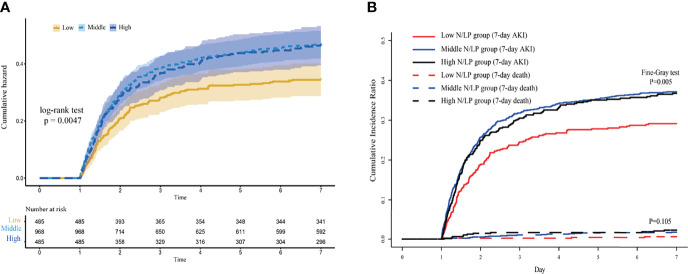
The cumulative incidence curve of sepsis acute kidney injury **(AKI)** plotted by **(A)** Kaplan-Meier method; **(B)** and competing risk model. AKI was diagnosed based on serum creatinine and urine output criteria.

AKI occurred in 680 (35.1%) of 1938 septic patients within seven days of ICU admission. As displayed in **Table 1**, the incidence of AKI varied significantly within each N/LP group. As compared to the low N/LP group, the middle and high N/LP groups had a significantly increased incidence of AKI (*P*<0.001) (**Figure 2A**). Additionally, the cumulative incidence curve calculated using the K-M method and the log-rank test result (*P*=0.0047) (**Figure 3A**) all followed the same trend as the above-mentioned founding. **Table 2** summarized the results of Cox proportional hazard models. Three statistical models with various adjusted confounders revealed that the hazard ratio (HR) and 95% confidence interval (CI) for both middle and high groups were larger than 1.0, indicating a higher incidence of AKI than the low N/LP group. In Model 2, after adjusting for all sixteen covariates, the HR (95%CI) for the middle and high groups was 1.30 (1.07, 1.58) (*P*=0.008) and 1.27 (1.02, 1.59) (*P*=0.034), respectively. It is important to note that the risk of AKI did not vary between the middle and high groups.

**Table 2:** The results of Cox proportional hazards models and competing risk analyses.

Group		Model 1			Model 2			Model3	
		HR (95% CI)	*P*		HR (95% CI)	*P*		HR (95% CI)	*P*
**Cox proportional hazard models**									
Low N/LP group		Ref	1		Ref	1		Ref	1
Middle N/LP group		1.37 (1.13, 1.66)	0.001		1.30 (1.07, 1.58)	0.008		1.31 (1.08, 1.59)	0.007
High N/LP group		1.34 (1.08, 1.68)	0.009		1.27 (1.02, 1.59)	0.034		1.28 (1.03, 1.60)	0.029
**Competing risk analyses**									
Low N/LP group		Ref	1		Ref	1		Ref	1
Middle N/LP group		1.36 (1.12, 1.66)	0.002		1.30 (1.07, 1.58)	0.008		1.30 (1.07, 1.58)	0.008
High N/LP group		1.33 (1.07, 1.66)	0.011		1.26 (1.01, 1.58)	0.038		1.27 (1.02, 1.58)	0.036

Model 1: univariate analysis; Model 2: adjusted for age, gender, initial SAPS II, SOFA scores excluding coagulation system, Charlson comorbidity index, serum AG, serum bicarbonate, glucose, serum potassium, serum sodium, serum chloride, BUN, SCr, the use of vasoactive medication, CRRT, and invasive-MV; Model 3: adjusted for age, serum chloride, SCr, sodium, SOFA scores excluding coagulation system, vasoactive medication, and invasive-MV; SOFA, Sequential Organ Failure Assessment; SAPS II, Simplified acute physiology score II; AKI, Acute kidney injury; CRRT, Continuous Renal Replacement Therapy; MV, machine ventilation; SCr, Serum Creatinine; BUN, Blood Urea Nitrogen; N/LP, Neutrophil-to-Lymphocyte Platelet.

On the other hand, only 31 (1.60%) individuals died during the first week after ICU admission without developing AKI. Additionally, the *Fine-Gray* test revealed that an early death was not a significant competing risk factor for the development of AKI (*P*=0.105). Thus, the cumulative incidence curve and trend of the competing-risks model were similar to those plotted by the *K-M* method (**Figure 3B**). **Table 2** presents the results of univariate and multivariate *Fine-Gray* competing-risks regression models. Moreover, no significant differences between competing-risks and Cox proportional hazard models were observed. Then, these findings revealed that an elevated initial N/LP level was related with an increased risk of early AKI in septic patients.

### The Relationship Between N/LP Ratios and Severe AKI

Among 680 sepsis patients with S-AKI, 260 (38.24%), 339 (49.85%), and 81 (11.91%) patients were diagnosed as stage 1, stage 2, and stage 3 based on both SCr and UO criteria. As illustrated in **Figure 2B**, severe AKI (stages 2 and 3) accounted for a greater proportion of the groups with a middle or high N/LP ratio. Besides, as the N/LP ratio increased, the proportion of patients with severe AKI risen substantially (**Figure 2C**).

**Figure 4:**
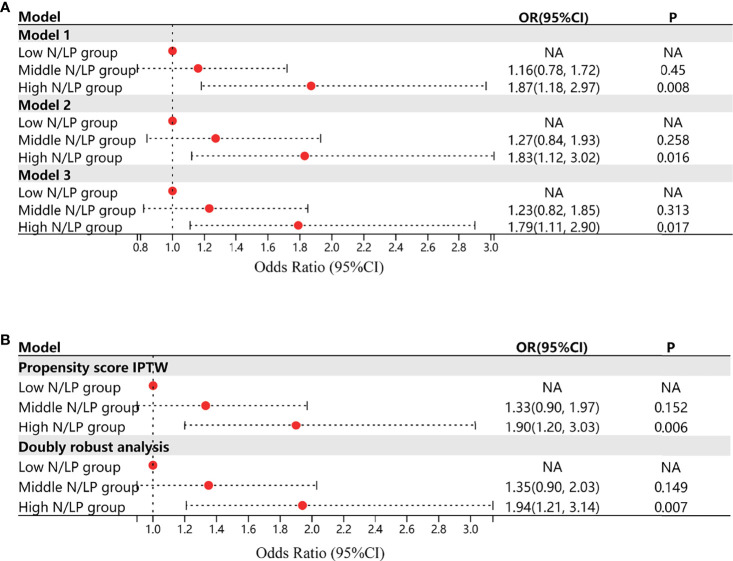
**(A)** The results of univariate and multivariate logistic regression when analysed the influence of varying N/LP levels on the occurrence of severe AKI in septic patients; Model 1 represented the univariate analysis; Model 2 represented the multivariate analysis adjusting all covariates; Model 3 represented the multivariate analysis adjusting gender, serum bicarbonate, serum chloride, serum creatinine, serum sodium, SOFA scores excluding coagulation system, Charlson comorbidity index based on the results of stepwise backward approach and collinearity analysis; **(B)** the results of univariate logistic analysis after inverse probability treatment weighting and the double robust estimation. Acute kidney injury was diagnosed based on serum creatinine and urine output criteria.

**Figure 5:**
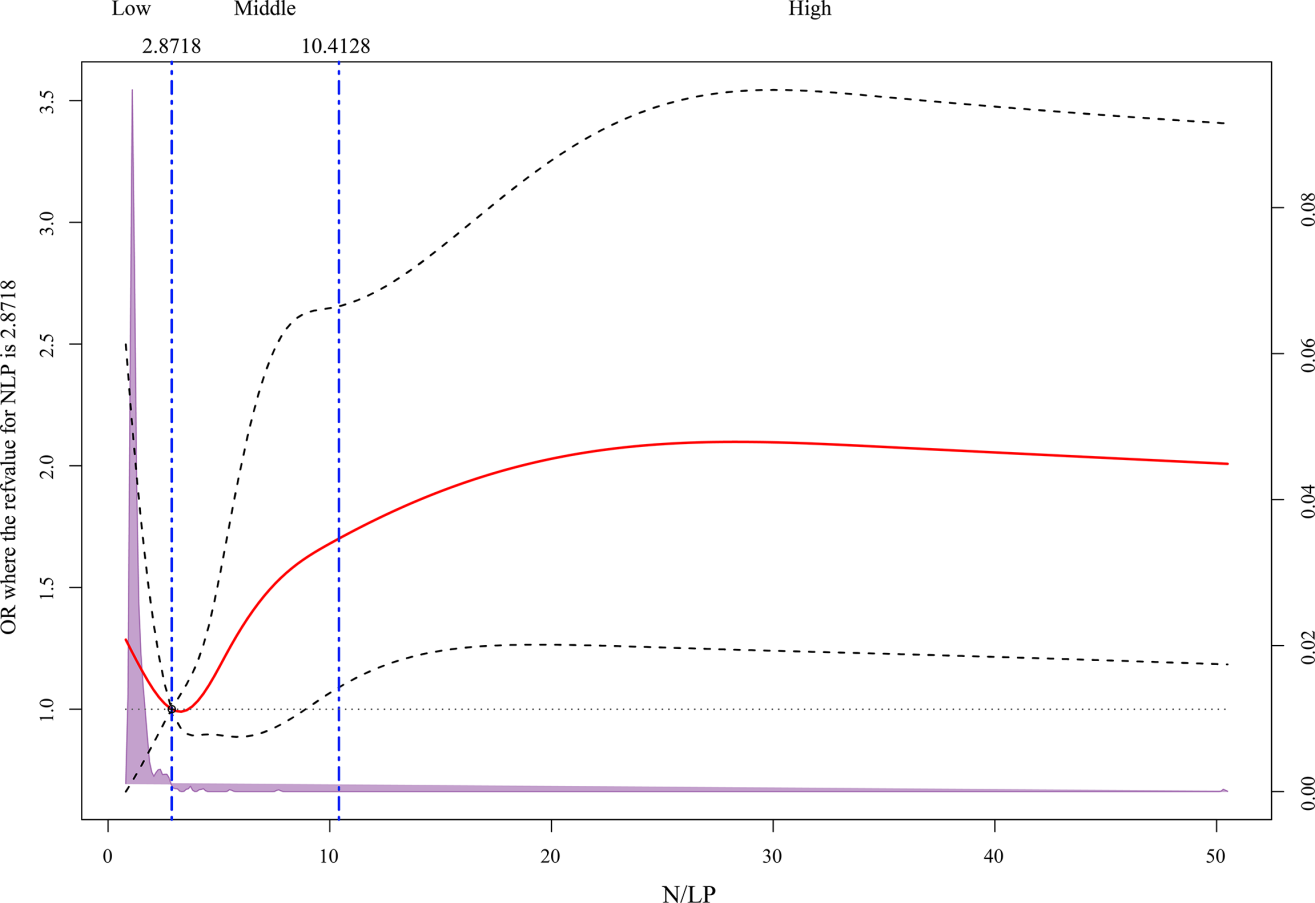
Multivariable adjusted odds ratios for severe acute kidney injury (AKI) occurrence according to initial N/LP on a continuous scale. Solid red lines were multivariable-adjusted odds ratios, with dashed bold lines showing 95% confidence intervals derived from restricted cubic spline regressions with five knots. Reference lines for no association were indicated by the black dashed lines at a hazard ratio of 1.0 and the reference knot setted at 2.8718. Purple regions indicated the fraction of the population with different levels of N/LP. All 16 covariates were adjusted. AKI and corresponding stage were diagnosed based on serum creatinine and urine output criteria.

The influence of varying N/LP levels on the occurrence of severe AKI in septic patients was then investigated by univariate and multivariate logistic regression. Each of the three models demonstrated a similar tendency (**Figure 4A**). When compared to the low N/LP group, a significant difference in the risk of severe AKI occurred only in the high N/LP group (aOR 1.83; 95%CI 1.12, 3.03), despite the fact that the odds ratio (OR) increased. RCS drew the N/LP dose-response curve and determined that the OR and 95%CI were greater than the dashed line on the Y-axis (Y = 1) only if the N/LP was more than around 9.5. (**Figure 5**). This finding was also consistent with logistic regression results.

### Double Robust Estimation

Prior to performing double robust estimation, we conducted IPTW to balance the baseline among three N/LP groups. **Supplementary Figure 1** shown the SMD of all 16 covariates before and after propensity score matching. The serious disequilibrium problem in original data has been well-resolved by IPTW based on multinomial logistic regression.

**Figure 6:**
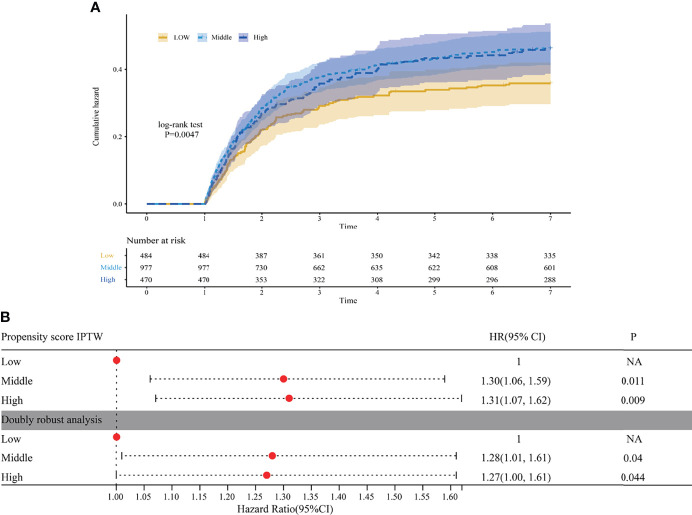
**(A)** Cumulative incidence curve of sepsis acute kidney injury (S-AKI) in each N/LP groups were plotted by Kaplan-Meier method after inverse probability treatment weighting (IPTW); **(B)** the results of univariate Cox proportional hazard model after IPTW and double robust estimation regressed all 16 covariates. AKI was diagnosed based on serum creatinine and urine output criteria.

Regarding S-AKI incidence, whether K-M methods or univariate Cox analysis after IPTW or double robust estimation regressed all 16 covariates included in the IPTW, a significantly increased risk of S-AKI was observed in middle and high N/LP groups compared with the low group (**Figure 6**). However, no difference was observed between the middle and high N/LP groups. When comparing the high N/LP group with the low N/LP group, univariate logistic analysis showed a nearly doubled risk of severe AKI (OR 1.90; 95%CI 1.20, 3.03) after IPTW. And, the double robust estimation adjusted for all 16 covariates also yielded a similar result (OR 1.94; 95%CI 1.21, 3.14) (**Figure 4B**).

### Subgroup Analysis

**Tables 3**, **4** summarized the results of subgroup analysis. Interaction tests with age, gender, septic shock, SOFA, SAPS II score, and Charlson comorbidity index were all non-significant for the risk of AKI (*P*=0.878, 0.674, 0.22, 0.455, 0.553, and 0.742) and severe AKI (*P*=0.213, 0.634, 0.355, 0.877, 0.543, and 0.674). Even so, we noticed that higher N/LP levels had a considerable impact on elder, male, septic shock patients, and those with poor health condition.

**Table 3:** Subgroup analysis regarding the influence of different N/LP level in the S-AKI occurrence. .

Subgroups	No.AKI/No.patients	Low N/LP group	Middle N/LP group	*P1*	High N/LP group	*P2*	*P* for interaction
**Age**							0.878
<65	303/920	Ref	1.20 (0.90, 1.61)	0.217	1.20 (0.85, 1.70)	0.302	
>=65	375/1018	Ref	1.40 (1.08, 1.83)	0.012	1.55 (1.14, 2.04)	0.005	
**Gender**							0.674
Female	288/884	Ref	1.27 (0.95, 1.69)	0.108	1.28 (0.90, 1.82)	0.164	
Male	390/1054	Ref	1.36 (1.04, 1.78)	0.026	1.42 (1.07, 1.89)	0.017	
**Septic shock**							0.22
NO	297/1074	Ref	1.14 (0.88, 1.48)	0.306	1.06 (0.78, 1.45)	0.705	
YES	381/864	Ref	1.51 (1.11, 2.05)	0.008	1.60 (1.13, 2.27)	0.008	
**SOFA_exclude_platelet**							0.455
<5	243/971	Ref	1.24 (0.91, 1.69)	0.182	1.25 (0.86, 1.83)	0.242	
>=5	435/967	Ref	1.31 (1.01, 1.67)	0.04	1.33 (1.12, 1.75)	0.015	
**SAPS II**							0.553
<35	262/928	Ref	1.14 (0.84, 1.54)	0.407	1.22 (0.84, 1.76)	0.303	
>=35	416/1010	Ref	1.39 (1.07, 1.81)	0.013	1.42 (1.11, 1.96)	0.007	
**Charlson comorbidity index**							0.742
<5	250/780	Ref	1.30 (0.94, 1.78)	0.108	1.26 (0.85, 1.86)	0.25	
>=5	428 /1158	Ref	1.29 (1.01, 1.66)	0.044	1.37(1.05, 1.81)	0.009	

P1: Middle N/LP group vs Low N/LP group; P2: High N/LP group vs Low N/LP group;

S-AKI, Sepsis associated acute kidney injury; N/LP, Neutrophil-to-Lymphocyte Platelet; SOFA, Sequential Organ Failure Assessment; SAPS II, Simplified acute physiology II.

**Table 4:** Subgroup analysis regarding the influence of different N/LP level in the severe AKI occurrence.

Subgroups	No.AKI/No.patients	Low	Middle	*P1*	High	*P2*	*P* for interaction
		N/LP group	N/LP group		N/LP group		
**Age**							0.213
<65	188/305	Ref	0.86( 0.46, 1.61)	0.646	1.35 (0.64, 2.88)	0.428	
>=65	232/375	Ref	1.52 (0.86, 2.71)	0.153	2.13 (1.07, 4.28)	0.032	
**Gender**							0.634
Female	193/289	Ref	1.07 (0.57, 2.02)	0.835	1.16 (0.53, 2.55)	0.706	
Male	227/391	Ref	1.43 (0.80, 2.57)	0.228	2.49 (1.27, 4.93)	0.008	
**Septic shock**							0.355
NO	188/298	Ref	1.41 (0.72, 2.73)	0.316	1.18 (0.55, 2.53)	0.66	
YES	232/382	Ref	1.17 (0.67, 2.04)	0.541	2.69 (1.36, 5.43)	0.005	
**SOFA_exclude_platelet**							0.877
<5	145/244	Ref	1.49 (0.75, 2.99)	0.259	2.11 (0.88, 5.21)	0.1	
>=5	275/436	Ref	1.18 (0.69, 2.02)	0.547	1.69 (1.09, 3.14)	0.025	
SAPS II							0.543
<35	151/263	Ref	0.73 (0.38, 1.38)	0.335	1.61 (0.72, 3.67)	0.253	
>=35	269/417	Ref	1.91 (1.08, 3.38)	0.026	2.16 (1.15, 4.12)	0.018	
**Charlson comorbidity index**							0.674
<5	138/251	Ref	0.79 (0.40, 1.56)	0.504	1.33 (0.58, 3.10)	0.498	
>=5	282/429	Ref	1.79 (1.04, 3.09)	0.036	2.23 (1.18, 4.23)	0.014	

P1, Middle N/LP group vs Low N/LP group; P2m High N/LP group vs Low N/LP group; the severe AKI refers to the stage 2 and stage 3 AKI;

AKI, Acute kidney injury; N/LP, Neutrophil-to-Lymphocyte Platelet; SOFA, Sequential Organ Failure Assessment; SAPS II, Simplified acute physiology II.

### Sensitivity Analysis

In sensitivity analyses, SCr criteria or UO criteria were separately used for AKI identification rather than in combination. When only SCr criteria was applied, we found that elevated N/LP levels would still increased the risk of S-AKI (**Supplementary Figure 2**); however, statistical significance merely appeared in the high N/LP groups, whether proved by the univariate or multivariate Cox proportional hazard model or competing-risks model (**Supplementary Table 2**), or even the double robust analysis regressed all covariates after the IPTW (**Supplementary Figure 3** and **Supplementary Figure 4**). In contrast, the N/LP levels were no longer associated with the risk of severe AKI (**Supplementary Figure 5** and **Supplementary Figure 6**).

While under UO criteria, the results were consistent with those obtained by previous analysis based on two indicators (SCr and UO) combination. In brief, the septic patients with an initial N/LP level greater than 2.8718 have a higher incidence of AKI, and the risk of severe AKI also significantly increased when N/LP was greater than 10.4128 (aOR 2.30; 95%CI 1.31, 4.08). **Supplementary Figure 7** showed the cumulative incidence curve drawn by the K-M method and univariate competing-risks regression model about AKI occurrence. **Supplementary Figure 8** presented the differences between each N/LP group in the original and the IPTW cohort, and **Supplementary Table 3**, **Supplementary Figure 9** presented the results of the Cox proportional hazard model and double robust analysis. Furthermore, **Supplementary Figure 10** and **Supplementary Figure 11** assessed the effect of high N/LP on the risk of severe AKI at different adjusting levels.

The sensitivity analysis results indicated that the UO criteria might be more suitable than SCr criteria when exploring the connection between the initial N/LP level with the occurrence of S-AKI and severe AKI.

## Discussion

### Key Findings

In the present study, various methods, such as competing risks models and double robust estimation, have been employed to evaluate the association between initial N/LP levels with the incidence of S-AKI and severe AKI in sepsis patients. We found that elevated N/LP would lead to increases in the risk of S-AKI and severe AKI within 7 days after ICU admission. Furthermore, these influences above were strengthened among males, elder, septic shock patients, and those with a poor health conditions. Finally, high NLP was more strongly connected to the risk and severity of AKI in sepsis patients on the UO-based criteria than on the SCr-based AKI criteria.

### Comparisons With Previous Studies

S-AKI is a common complication in critical septic patients and is associated with high morbidity and mortality (21). Due to the difficulty in prevention, early recognition of S-AKI is essential for timely intervention and improving prognosis. According to recent evidence, inflammatory response, microvascular dysfunction, and metabolic reprogramming may be the underlying mechanisms responsible for causing S-AKI (22). Since sepsis triggers a systemic cytokine-chemokine response, it would result in an extensive activation and dysfunction of the immune system, manifested as neutrophilia and lymphocytopenia (23). Thus, the neutrophil-to-lymphocyte ratio (NLR), calculated from whole blood counts, was proposed as a surrogate indicator to reflect the relative relationship between the inflammatory response and immune status (24). Previous studies have demonstrated that NLR may be valuable for predicting the disease outcome in multiple diseases, such as cardiovascular disease (25, 26), cancer (27), and sepsis (28, 29).

Although NLR had been reported to predict the development of AKI in sepsis, its sensitivity and specificity were limited (24). The possible reason is the complex interaction between immune mechanisms, inflammatory cascade activation, and coagulation pathway disorders. Subsequently, these interactions would result in microvascular dysfunction, leukocyte/platelet activation, and microthrombi formation, ultimately inducing renal tubular epithelial cell injury (30). Because of the intimate association between coagulation and the inflammatory response, platelets have been considered a critical factor in the initiation and progression of AKI development in sepsis (31). Hence, N/LP, suggested as a surrogate indicator for NLR, can shed light on the relationship between systemic inflammation, immunity, and coagulation disorders comprehensively (13). Moreover, recent research presented that the levels of postoperative N/LP were significantly associated with AKI after abdominal and cardiovascular surgery (12, 13, 32). In addition, several retrospective studies have also indicated that a rising N/LP ratio is an efficient predictor of the risk of in-hospital mortality in patients with S-AKI and those undergoing emergency surgery (14, 33). However, no study has reported the relationship between N/LP and the occurrence and severity of AKI in sepsis patients. In the present study, we found that elevated N/LP in sepsis patients was associated with an increased risk of AKI. Furthermore, we also demonstrated that the risk of severe AKI (KDIGO stages 2 and 3) increased more than 2-fold when N/LP was over 10. These findings suggest that early N/LP elevation may serve as a potential predictor of the occurrence and severity of AKI in sepsis patients.

KDIGO guideline considers SCr and/or oliguria to have equal prognostic power for diagnosing AKI. Nevertheless, it is difficult to accurately obtain the baseline value when using the SCr criteria for AKI diagnosing due to the lack of uniform delineation criteria (34). UO is also insensitive and easily influenced by many factors (15). Several studies have shown poor consistency between SCr and UO criteria in AKI diagnosing and the corresponding staging (35–37). A sizeable single-center retrospective study with 32, 045 critically ill patients found that UO-based and SCr-based criteria have different diagnostic power for AKI (36). In another retrospective study of 6637 patients undergoing cardiac surgery, the incidence of AKI increased from 38.6% to 81.2% after considering UO criteria (37). Similarly, using the SCr criteria alone may miss approximately 20% of AKI patients and lead to AKI grade misclassification (34, 36). Bianchi et al. (15) proved that oliguria lasting longer than 12 hours (KDIGO stage 2 and 3) was significantly diagnostic of AKI in 15, 620 patients and was not accompanied by elevated SCr levels. In this study, high N/LP was more strongly associated with the risk of S-AKI and severe AKI among sepsis patients based on UO-based rather than SCr-based AKI criteria. A previous study found that enhanced monitoring of UO improved detection of AKI and reduced 30-day mortality in patients with AKI (38). Therefore, we suggest that enhanced monitoring of UO and N/LP may be more useful in guiding clinical decision-making in S-AKI, especially in some special populations (elder, male, septic shock, and patients with a poor health condition).

### Strengths and Limitations

This study has several strengths. The relationship between N/LP and the risk and severity of S-AKI was investigated for the first time, and the effect of N/LP on S-AKI was analyzed by Cox proportional risk model, competing risk model, and double robust estimation. The results are reliable and stable and provide a basis for clinical diagnosis and intervention in S-AKI. Notably, high N/LP based on UO criteria more strongly correlated with the risk of S-AKI and severe AKI in patients with sepsis compared with KDIGO AKI criteria. Thus, enhanced monitoring of UO and N/LP would be more helpful in guiding ICU physicians’ clinical decision-making regarding S-AKI. However, this study has several limitations. First, MIMIC-IV is a single-center database, and selection bias exists in this study, limiting our conclusions’ extrapolation. Second, we only discussed the influence of single indicator N/LP on the occurrence and development of S-AKI. Finally, our study only examined N/LP values within 24 hours of ICU admission in patients with sepsis and failed to evaluate the dynamic effect of N/LP, which was related to the absence of relevant information in the MIMIC database. In future studies, the predictive value of N/LP for S-AKI can be further evaluated by its dynamic changes.

## Conclusions

Early assessment and intervention are crucial for managing S-AKI patients in the ICU. An initial elevated N/LP level may induce the development of S-AKI and severe AKI within 7 days after ICU admission in septic patients. These influences were enhanced in elder, males, septic shock, and those with a poor health condition. Furthermore, high NLP was more strongly connected to the risk of S-AKI and severe AKI in sepsis patients on the UO-based AKI criteria than on the SCr-based criteria. Therefore, enhanced monitoring of UO and N/LP would be more helpful in guiding clinical decisions about S-AKI. Of course, the effectiveness of N/LP in guiding the treatment of AKI in sepsis needs to be further investigated.

## Data Availability Statement

The original contributions presented in the study are included in the article/**Supplementary Material**, further inquiries can be directed to the corresponding author/s.

## Ethics Statement

MIMIC-IV database is a publicly available anonymized database, approval for the ethical committee was not necessary.

## Author Contributions

XW and YM designed the study. LZ and LY extracted the data. XW, LZ and HT conducted data quality management and statistical analysis and drafted the manuscript. ZJ and HJ participated in the literature search. LH, XY and YM critically revised the manuscript. All authors contributed to the article and approved the submitted version.

## Funding

This study was supported by a research grant from the National Natural Science Foundation of China (No. 82072134) and the National Natural Science Foundation Youth Science Foundation (No. 81601661).

## Conflict of Interest

KS is employed by Daiichi Sankyo Co., Ltd.

The remaining authors declare that the research was conducted in the absence of any commercial or financial relationships that could be construed as a potential conflict of interest.

## Publisher’s Note

All claims expressed in this article are solely those of the authors and do not necessarily represent those of their affiliated organizations, or those of the publisher, the editors and the reviewers. Any product that may be evaluated in this article, or claim that may be made by its manufacturer, is not guaranteed or endorsed by the publisher.

## Supplementary Material

Supplementary Figure 1 SMD of all 16 covariables before and after IPTW in (A) 1938 included sepsis patients, (B) and 680 patients with acute kidney injury diagnosed by SCr and UO criteria. SMD, standardized mean difference; IPTW, inverse probability of treatment weighting; LR, logistic regression; SAPS II, simplified acute physiology score II; SOFA, sequential organ failure assessment; CRRT, continuous renal replacement therapy; BUN, blood urea nitrogen; SCr, serum creatinine; UO, urine output.

Supplementary Figure 2 The cumulative incidence curve of sepsis acute kidney injury (S-AKI) plotted by (A) Kaplan-Meier method, (B) and competing risk model. AKI is diagnosed based on serum creatinine criteria only.

Supplementary Figure 3 (A) The cumulative incidence curve of sepsis acute kidney injury (S-AKI) plotted by Kaplan-Meier method after inverse probability treatment weighting (IPTW); (B) the results of univariate Cox proportional hazard model after IPTW and double robust estimation regressed all 16 covariates. AKI is diagnosed based on serum creatinine criteria only.

Supplementary Figure 4 SMD of all 16 covariables before and after IPTW in sensitivity analysis; (A) all sepsis patients with SCr records; (B) those who fulfill the SCr criteria in acute kidney injury diagnosing. SMD, standardized mean difference; IPTW, inverse probability of treatment weighting; LR, logistic regression; SAPS II, simplified acute physiology score II; SOFA, sequential organ failure assessment; CRRT, continuous renal replacement therapy; BUN, blood urea nitrogen; SCr, serum creatinine.

Supplementary Figure 5 (A) The results of univariate and multivariate logistic regression. Model 1 represents the univariate analysis; Model 2 represents the multivariate analysis adjusting all covariates; Model 3 represents the multivariate analysis adjusting serum chloride, serum creatinine, SOFA scores excluding coagulation system, Charlson comorbidity index, the use of vasoactive medication, and invasive machine ventilation based on the results of stepwise backward approach and collinearity analysis; (B) the results of univariate logistic analysis after inverse probability treatment weighting and the double robust estimation. Acute kidney injury is diagnosed based on serum creatinine criteria only.

Supplementary Figure 6 Multivariable adjusted odds ratios for severe acute kidney injury (AKI) occurrence according to initial N/LP on a continuous scale by using restricted cubic splines analysis. AKI and corresponding stage are diagnosed based on serum creatinine criteria only.

Supplementary Figure 7 The cumulative incidence curve of sepsis acute kidney injury (S-AKI) plotted by (A) Kaplan-Meier method, and (B) competing risk model. AKI is diagnosed based on urine output criteria only.

Supplementary Figure 8 SMD of all 16 covariables before and after IPTW in sensitivity analysis based; (A) all sepsis patients with urine output (UO) records; (B) those who fulfill the UO criteria in acute kidney injury diagnosing. SMD, standardized mean difference; IPTW, inverse probability of treatment weighting; LR, logistic regression; SAPS II, simplified acute physiology score II; SOFA, sequential organ failure assessment; CRRT, continuous renal replacement therapy; BUN, blood urea nitrogen; UO, urine output.

Supplementary Figure 9 (A) The cumulative incidence curve of sepsis acute kidney injury (S-AKI) plotted by Kaplan-Meier method after inverse probability treatment weighting (IPTW); (B) the results of univariate Cox proportional hazard model after IPTW and double robust estimation regressed all 16 covariates. AKI is diagnosed based on urine output criteria only.

Supplementary Figure 10 (A) The results of univariate and multivariate logistic regression. Model 1 represents the univariate analysis; Model 2 represents the multivariate analysis adjusting all covariates; Model 3 represents the multivariate analysis adjusting gender, serum bicarbonate, serum sodium, SOFA scores excluding coagulation system, Charlson comorbidity index, and the use of vasoactive medication based on the results of stepwise backward approach and collinearity analysis; (B) the results of univariate logistic analysis after inverse probability treatment weighting and the double robust estimation. Acute kidney injury is diagnosed based on urine output criteria only.

Supplementary Figure 11 Multivariable adjusted odds ratios for severe acute kidney injury (AKI) occurrence according to initial N/LP on a continuous scale by using restricted cubic splines analysis. AKI and corresponding stage are diagnosed based on urine output criteria only.

## Abbreviations

N/LP, neutrophils to lymphocytes and platelets; NLR, neutrophil-to-lymphocyte ratio; AKI, acute kidney injury; ICU, intensive care unit; S-AKI, sepsis-associated acute kidney injury; RCS, restricted cubic splines; SCr, serum creatinine; UO, urine output; MIMIC-IV, Medical Information Mart for Intensive Care IV; ICD, International Classification of Diseases; WBC, white blood cell count; BUN, blood urea nitrogen; AG, anion gap; SAPS II, Simplify the Acute Physiological Scores II; SOFA, Sequential Organ Failure Assessment; CRRT, continuous renal replacement therapy; MV, mechanical ventilation; KDIGO, Kidney Disease: Improving Global Outcomes Clinical Practice Guidelines; VIF, variance inflation factors; IPTW, inverse probability treatment weighting; SMD, standardized mean difference; K-M, Kaplan-Meier.
