# Supplementary figures and images for: Influence of the Initial Neutrophils to Lymphocytes and Platelets Ratio on the Incidence and Severity of Sepsis-Associated Acute Kidney Injury: A Double Robust Estimation Based on a Large Public Database
Source: Front Immunol. 2022 Jul 12;13:925494. doi: 10.3389/fimmu.2022.925494 (PMC9320191; doi:10.3389/fimmu.2022.925494)

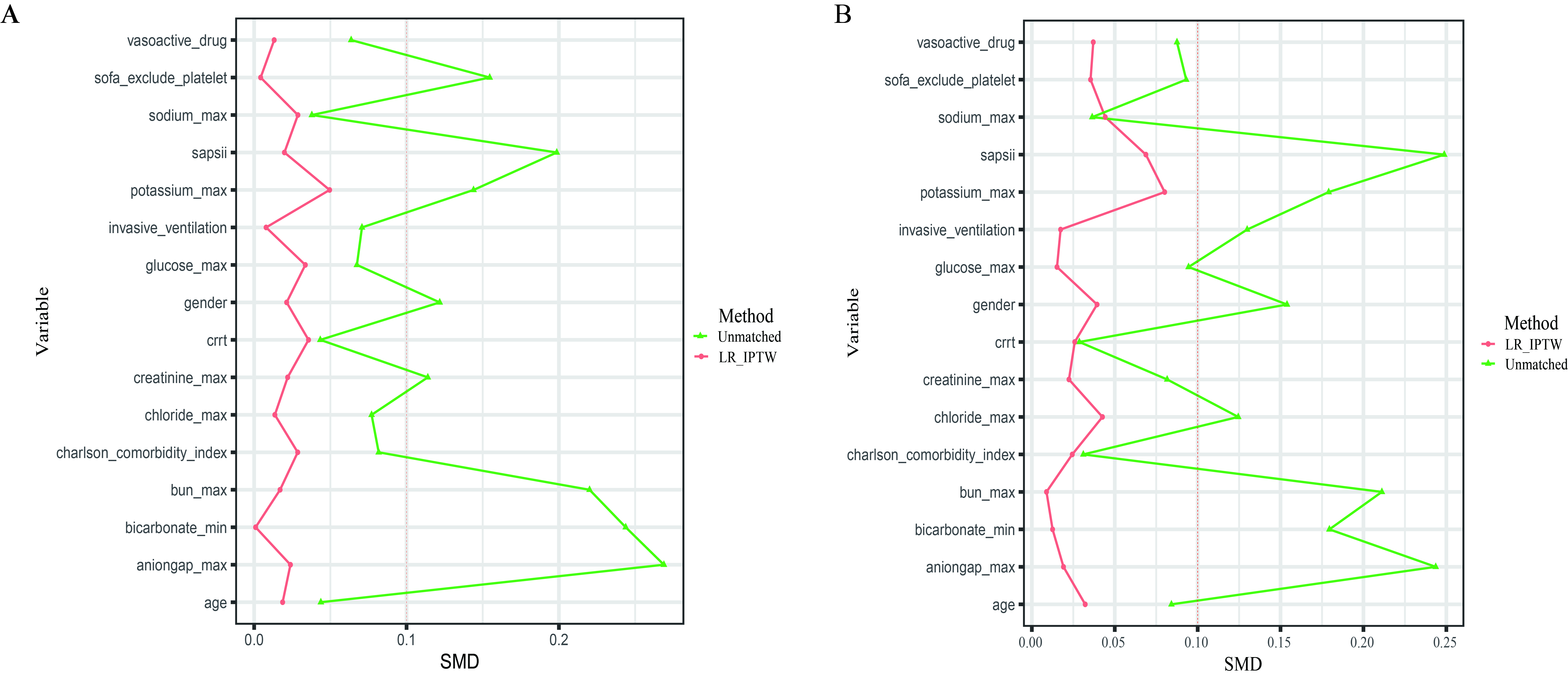

Supplement: Supplementary Figure 1 — SMD of all 16 covariables before and after IPTW in (A) 1938 included sepsis patients, (B) and 680 patients with acute kidney injury diagnosed by SCr and UO criteria. SMD, standardized mean difference; IPTW, inverse probability of treatment weighting; LR, logistic regression; SAPS II, simplified acute physiology score II; SOFA, sequential organ failure assessment; CRRT, continuous renal replacement therapy; BUN, blood urea nitrogen; SCr, serum creatinine; UO, urine output. [file Image_1.tif]

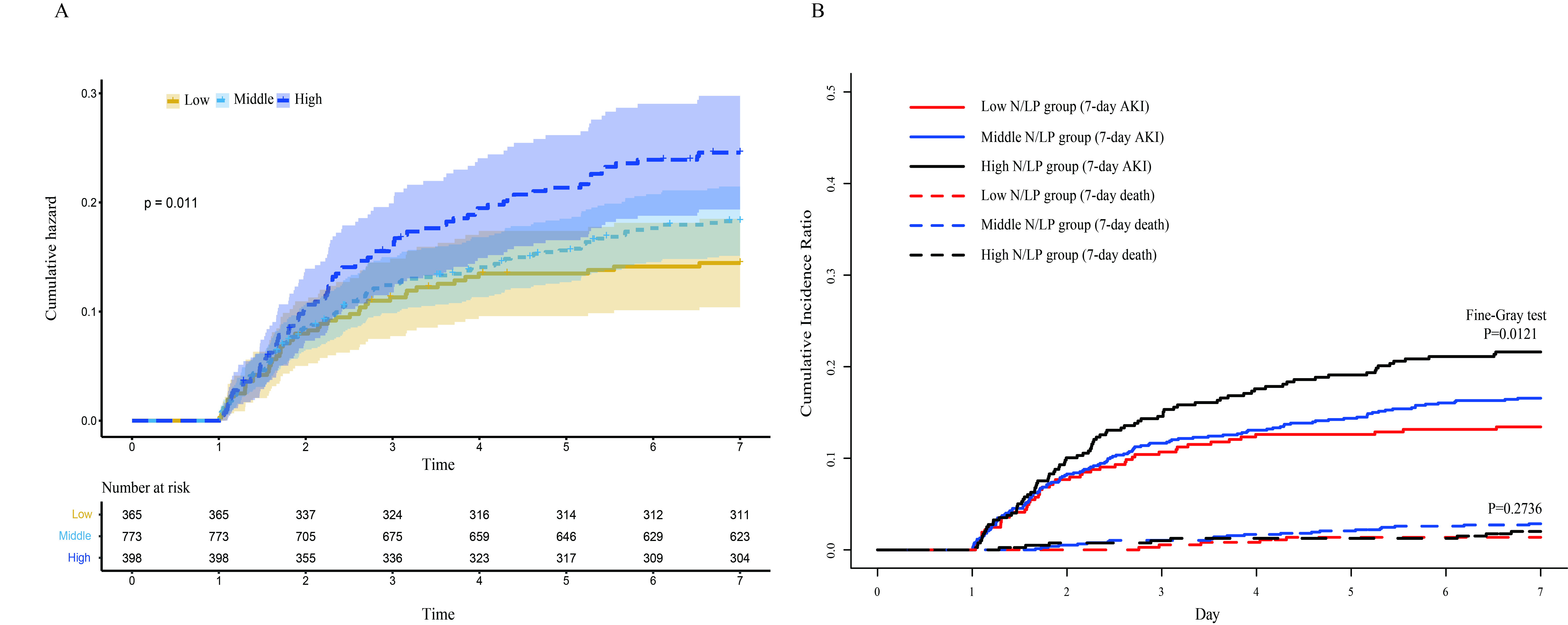

Supplement: Supplementary Figure 2 — The cumulative incidence curve of sepsis acute kidney injury (S-AKI) plotted by (A) Kaplan-Meier method, (B) and competing risk model. AKI is diagnosed based on serum creatinine criteria only. [file Image_2.tif]

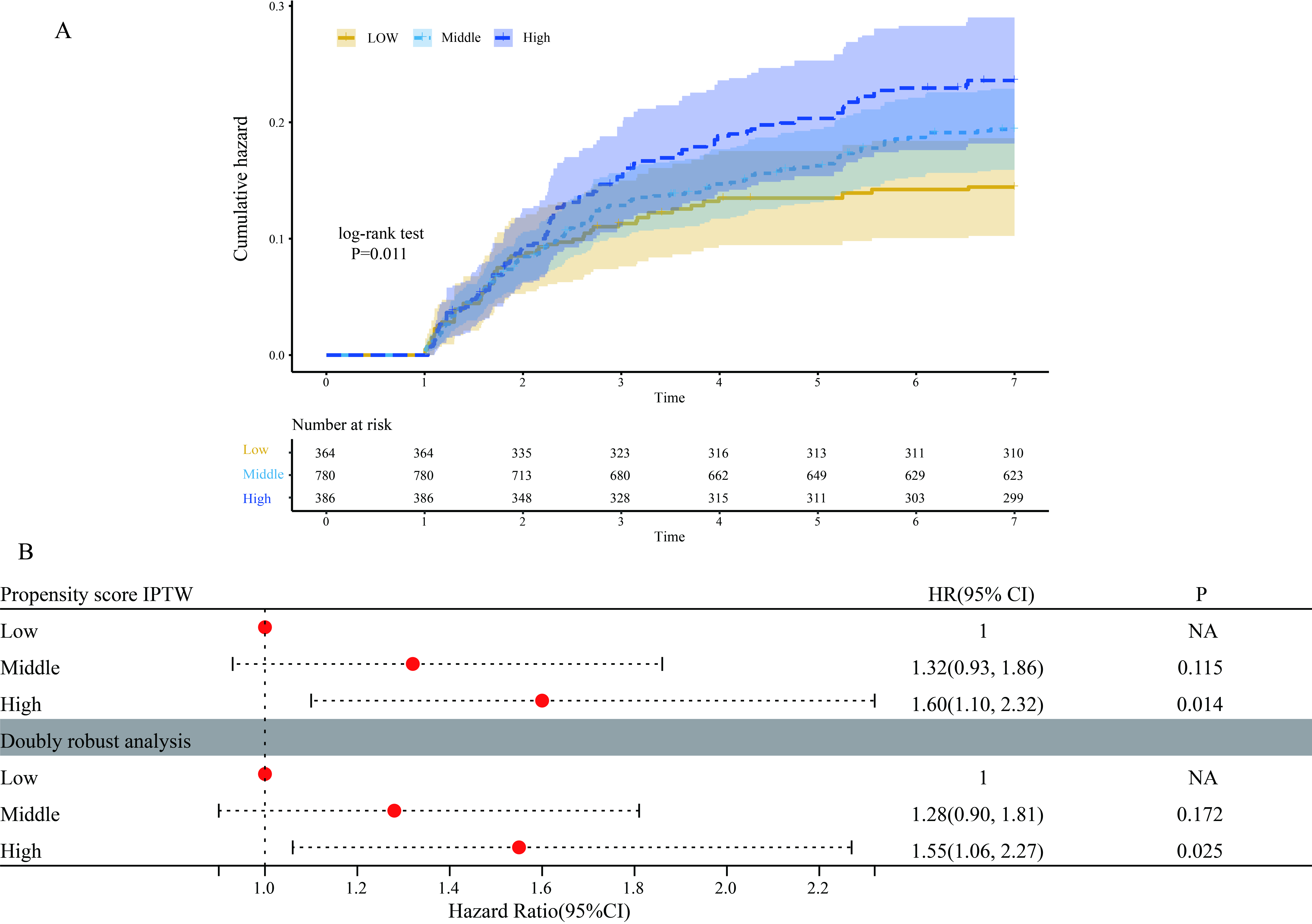

Supplement: Supplementary Figure 3 — (A) The cumulative incidence curve of sepsis acute kidney injury (S-AKI) plotted by Kaplan-Meier method after inverse probability treatment weighting (IPTW); (B) the results of univariate Cox proportional hazard model after IPTW and double robust estimation regressed all 16 covariates. AKI is diagnosed based on serum creatinine criteria only. [file Image_3.tif]

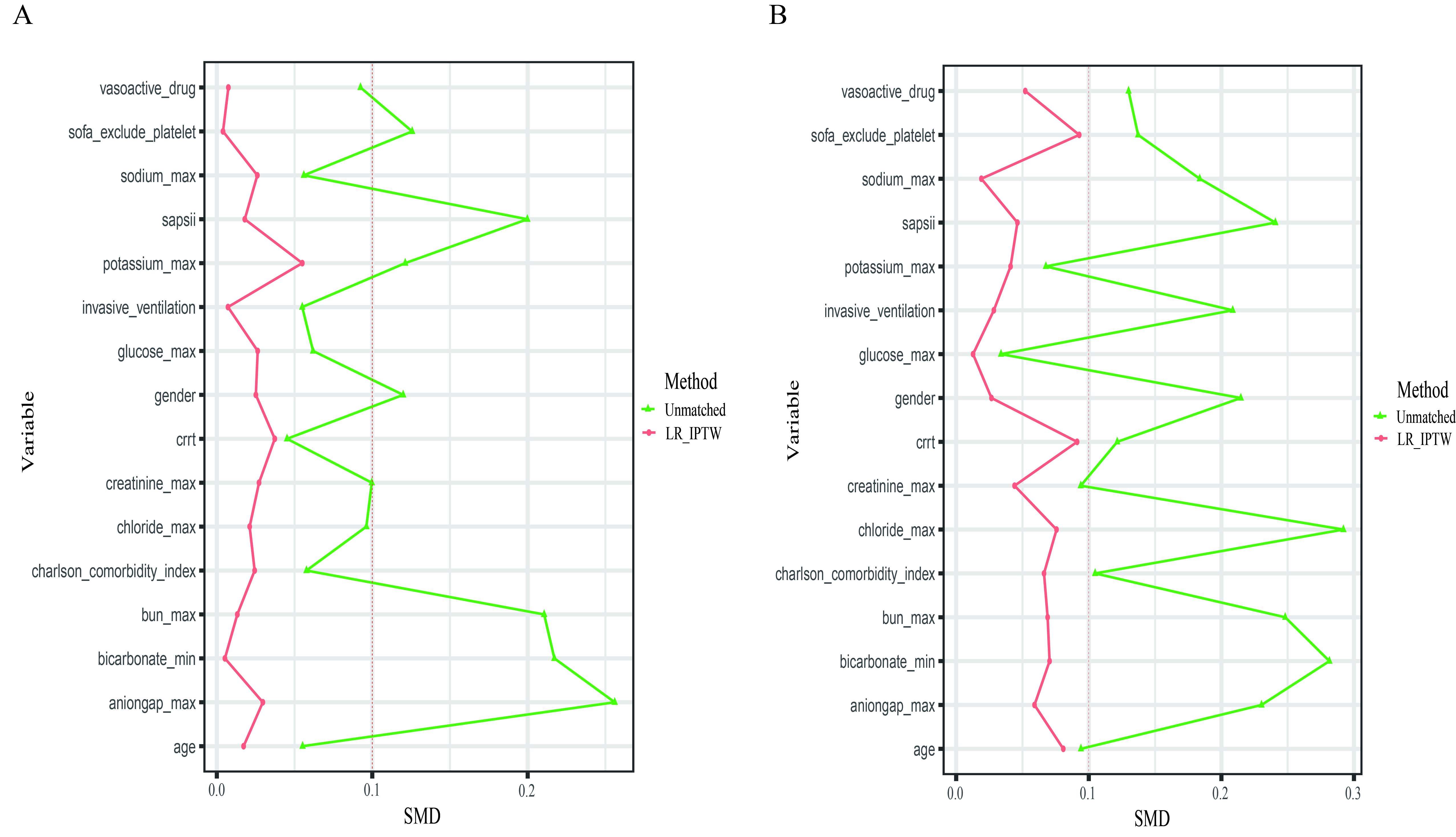

Supplement: Supplementary Figure 4 — SMD of all 16 covariables before and after IPTW in sensitivity analysis; (A) all sepsis patients with SCr records; (B) those who fulfill the SCr criteria in acute kidney injury diagnosing. SMD, standardized mean difference; IPTW, inverse probability of treatment weighting; LR, logistic regression; SAPS II, simplified acute physiology score II; SOFA, sequential organ failure assessment; CRRT, continuous renal replacement therapy; BUN, blood urea nitrogen; SCr, serum creatinine. [file Image_4.tif]

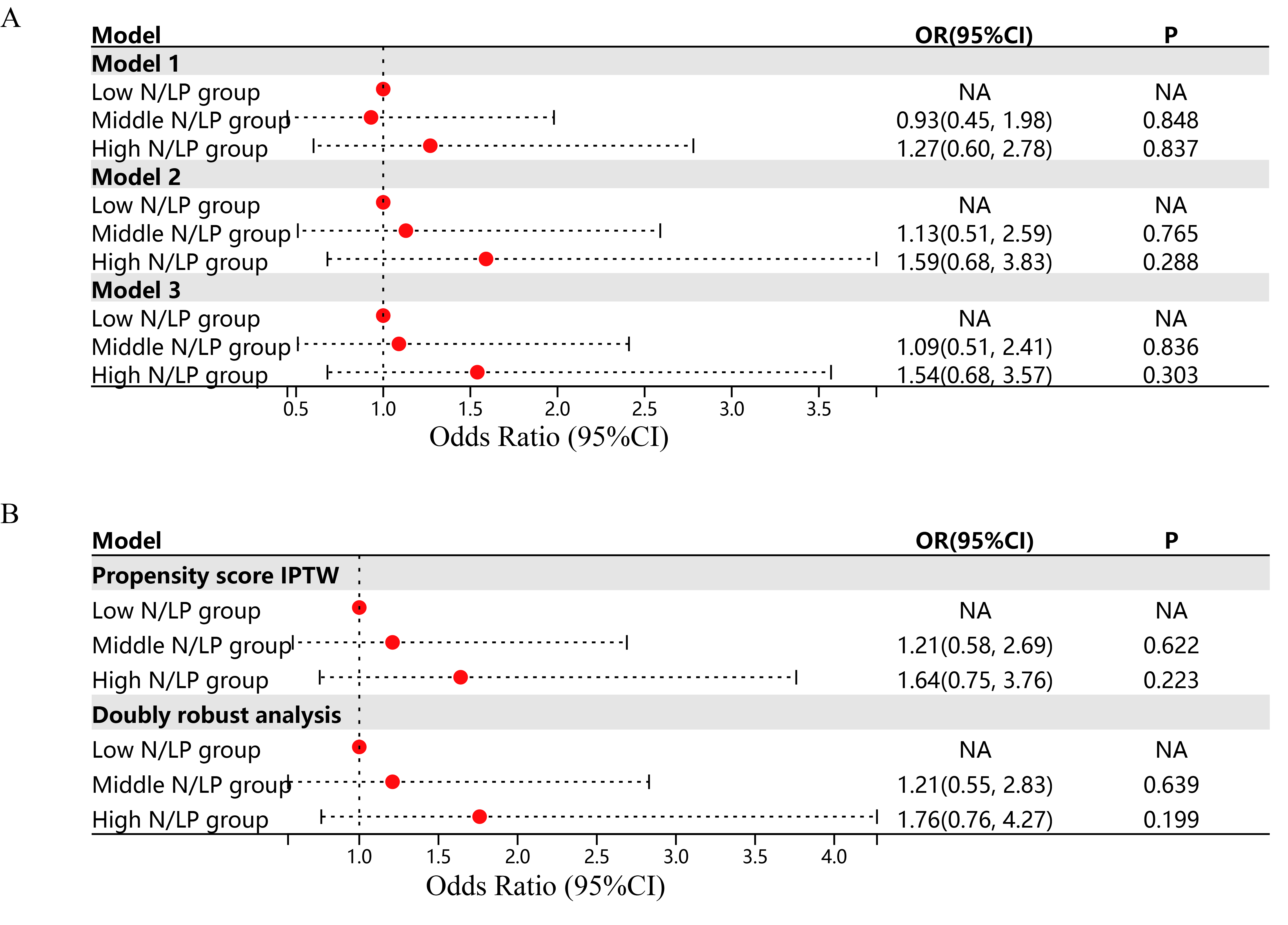

Supplement: Supplementary Figure 5 — (A) The results of univariate and multivariate logistic regression. Model 1 represents the univariate analysis; Model 2 represents the multivariate analysis adjusting all covariates; Model 3 represents the multivariate analysis adjusting serum chloride, serum creatinine, SOFA scores excluding coagulation system, Charlson comorbidity index, the use of vasoactive medication, and invasive machine ventilation based on the results of stepwise backward approach and collinearity analysis; (B) the results of univariate logistic analysis after inverse probability treatment weighting and the double robust estimation. Acute kidney injury is diagnosed based on serum creatinine criteria only. [file Image_5.tif]

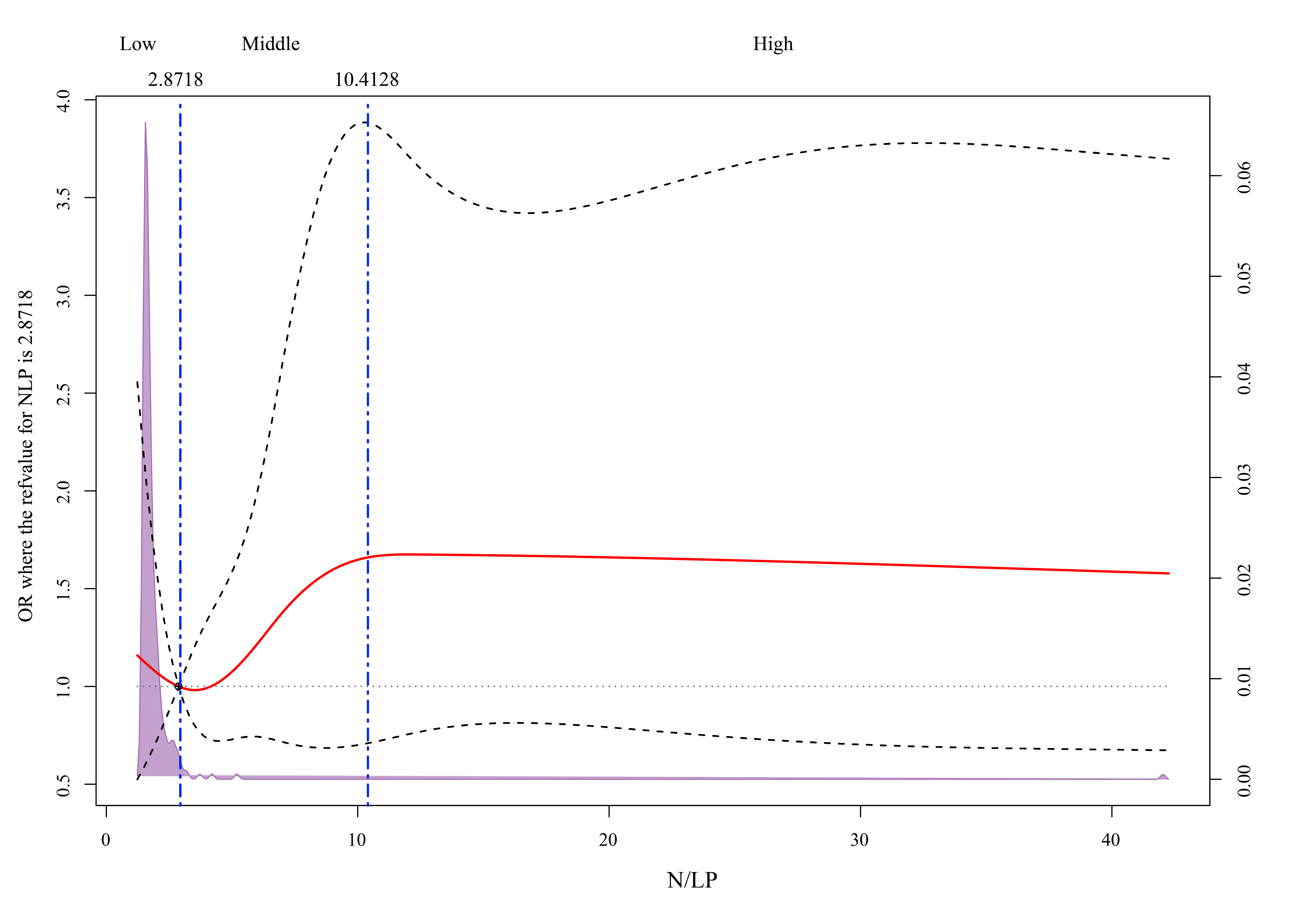

Supplement: Supplementary Figure 6 — Multivariable adjusted odds ratios for severe acute kidney injury (AKI) occurrence according to initial N/LP on a continuous scale by using restricted cubic splines analysis. AKI and corresponding stage are diagnosed based on serum creatinine criteria only. [file Image_6.tif]

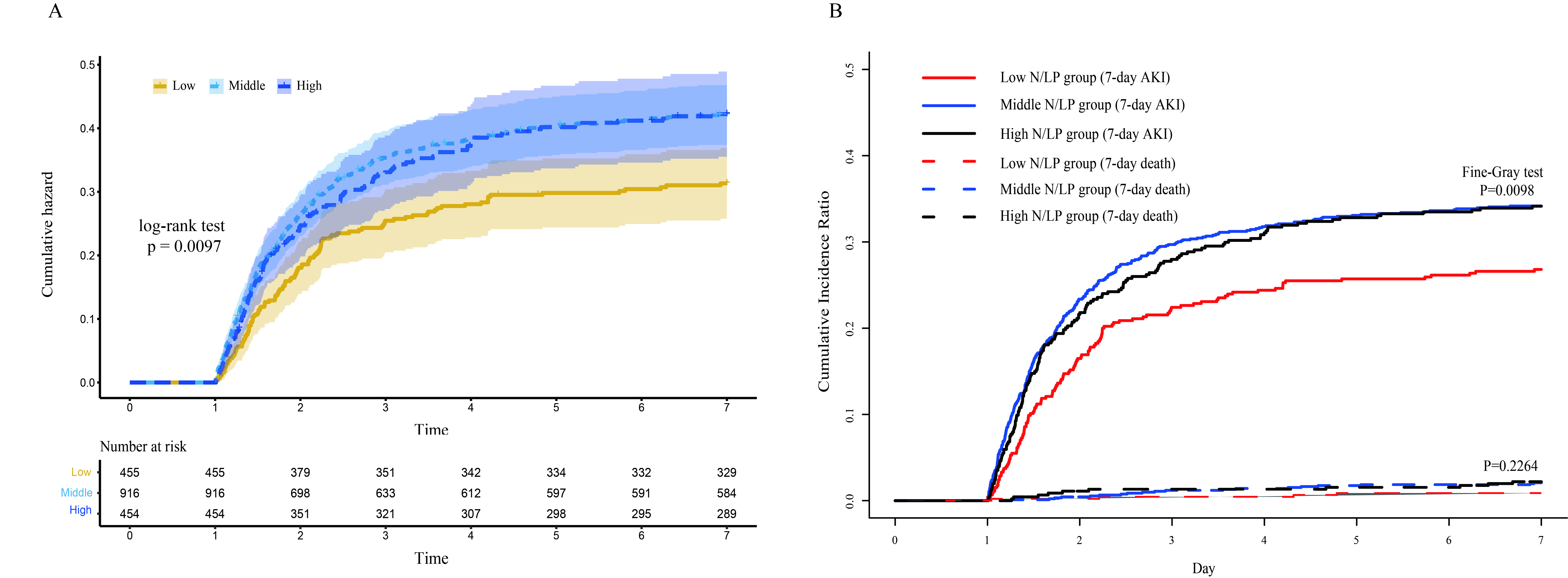

Supplement: Supplementary Figure 7 — The cumulative incidence curve of sepsis acute kidney injury (S-AKI) plotted by (A) Kaplan-Meier method, and (B) competing risk model. AKI is diagnosed based on urine output criteria only. [file Image_7.tif]

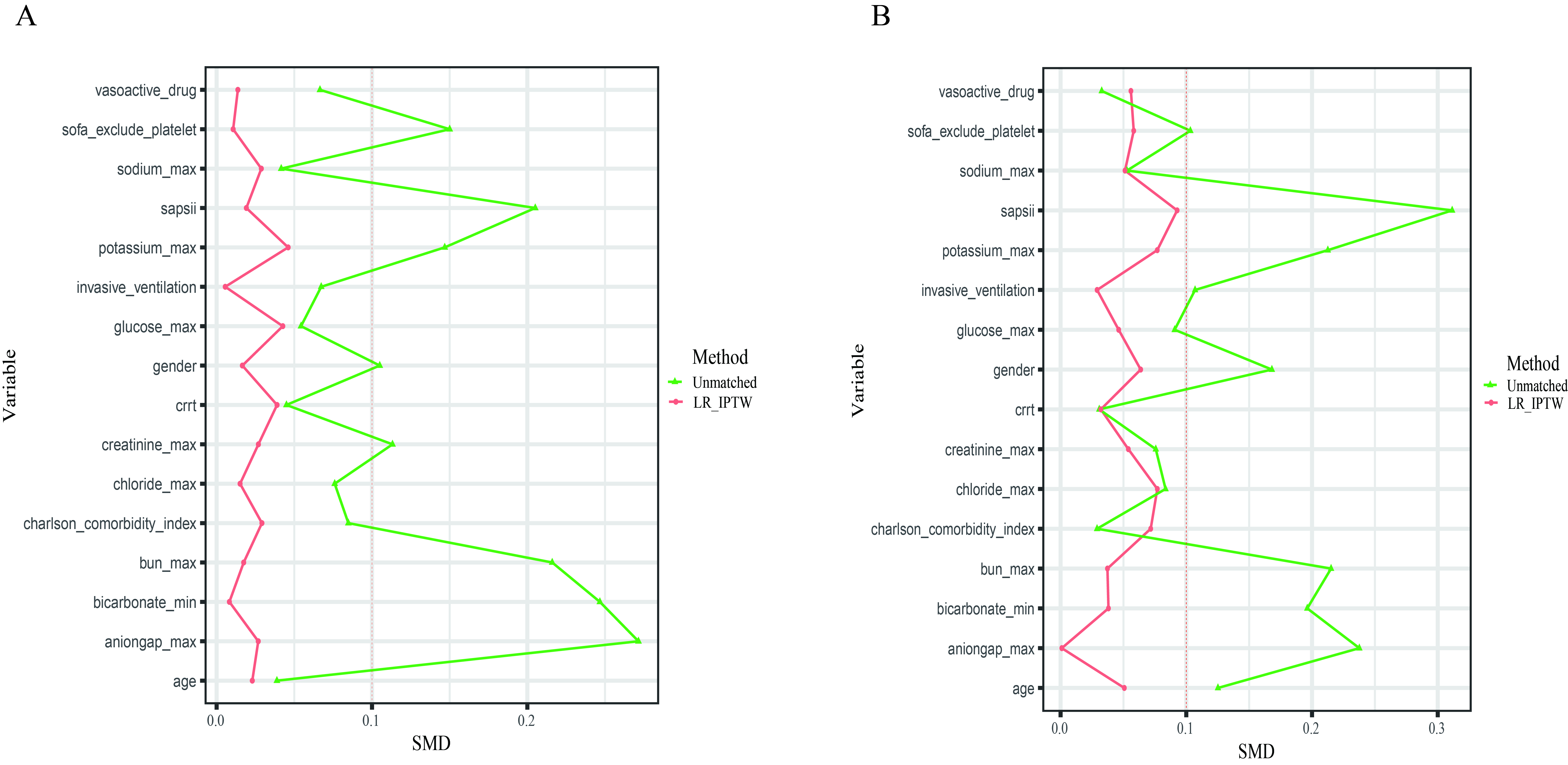

Supplement: Supplementary Figure 8 — SMD of all 16 covariables before and after IPTW in sensitivity analysis based; (A) all sepsis patients with urine output (UO) records; (B) those who fulfill the UO criteria in acute kidney injury diagnosing. SMD, standardized mean difference; IPTW, inverse probability of treatment weighting; LR, logistic regression; SAPS II, simplified acute physiology score II; SOFA, sequential organ failure assessment; CRRT, continuous renal replacement therapy; BUN, blood urea nitrogen; UO, urine output. [file Image_8.tif]

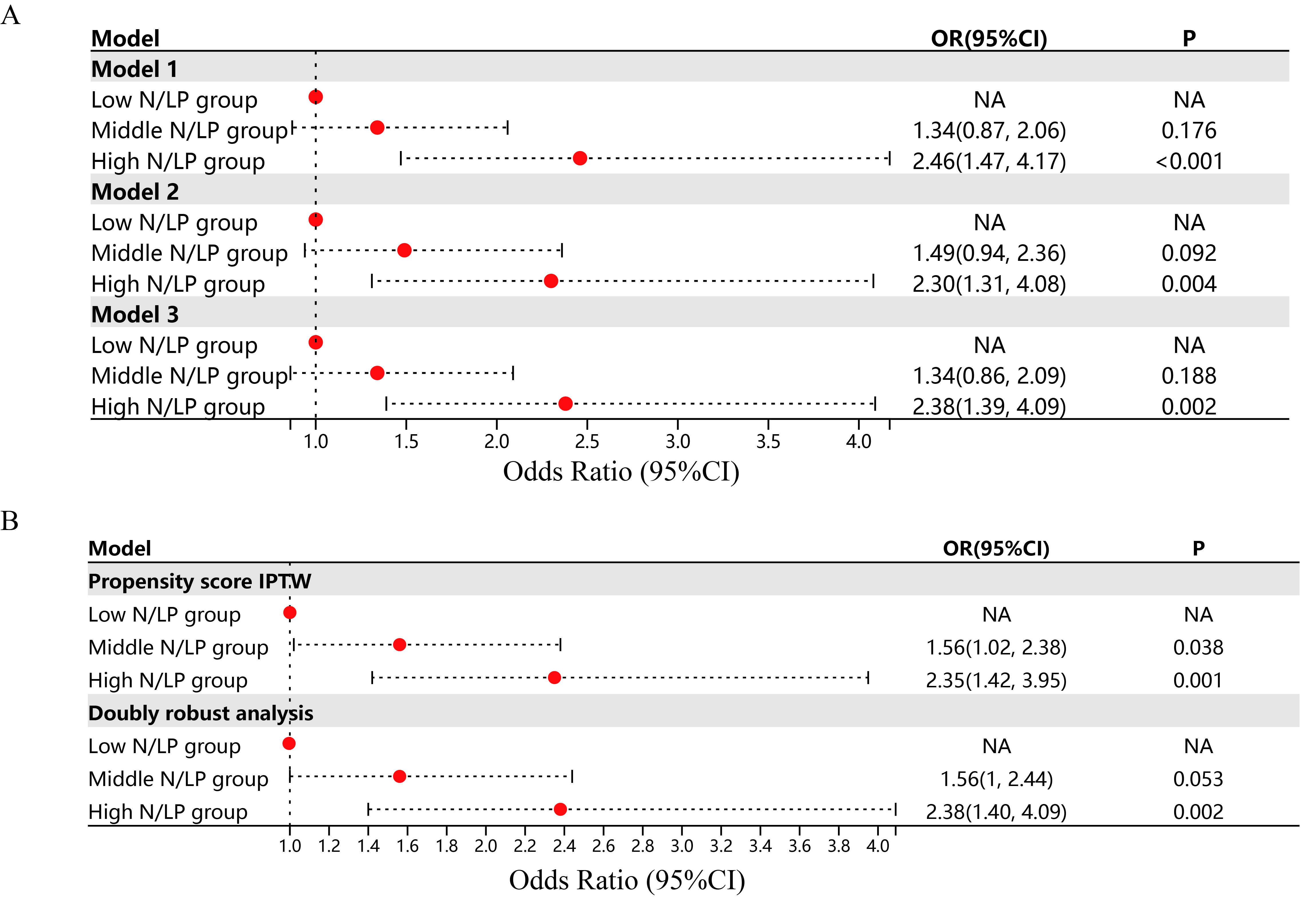

Supplement: Supplementary Figure 10 — (A) The results of univariate and multivariate logistic regression. Model 1 represents the univariate analysis; Model 2 represents the multivariate analysis adjusting all covariates; Model 3 represents the multivariate analysis adjusting gender, serum bicarbonate, serum sodium, SOFA scores excluding coagulation system, Charlson comorbidity index, and the use of vasoactive medication based on the results of stepwise backward approach and collinearity analysis; (B) the results of univariate logistic analysis after inverse probability treatment weighting and the double robust estimation. Acute kidney injury is diagnosed based on urine output criteria only. [file Image_10.tif]

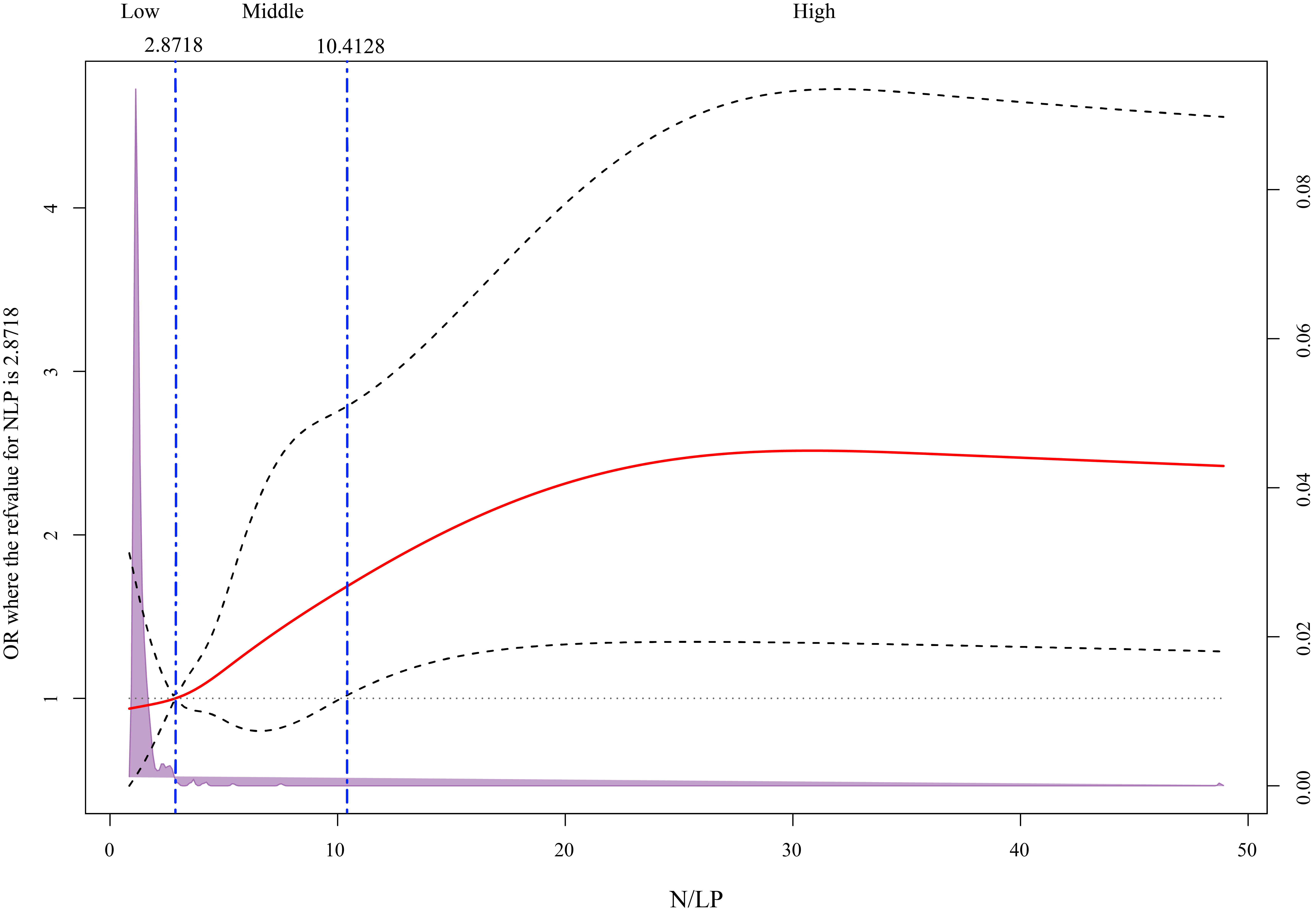

Supplement: Supplementary Figure 11 — Multivariable adjusted odds ratios for severe acute kidney injury (AKI) occurrence according to initial N/LP on a continuous scale by using restricted cubic splines analysis. AKI and corresponding stage are diagnosed based on urine output criteria only. [file Image_11.tif]
