# Supplementary material for: Influence of the Initial Neutrophils to Lymphocytes and Platelets Ratio on the Incidence and Severity of Sepsis-Associated Acute Kidney Injury: A Double Robust Estimation Based on a Large Public Database
Source: Front Immunol. 2022 Jul 12;13:925494. doi: 10.3389/fimmu.2022.925494 (PMC9320191; doi:10.3389/fimmu.2022.925494)
Supplement: Supplementary file 12 [file Table_1.docx]

Supplementary Table 1The characteristics of patients in whom AKI occurred (S-AKI group) and patients in whom AKI did not occur (non S-AKI group)

| **Variables** | **Non S-AKI (n = 1258)** | **S-AKI (n = 680)** | ***P*** |
| --- | --- | --- | --- |
| **Age (year)** | 65.48 (53.58, 77.96) | 66.77 (56.21, 79.35) | 0.035 |
| **Gender (%)** |  |  | 0.048 |
| Female | 663 (53) | 391 (57) |  |
| Male | 595 (47) | 289 (42) |  |
| **Ethnicity (%)** |  |  | <0.001 |
| AMERICAN INDIAN/ALASKA NATIVE | 1(0.05%) | 6(0.31%) |  |
| ASIAN | 25(1.29%) | 75(3.87%) |  |
| BLACK/AFRICAN AMERICAN | 46(2.37%) | 160(8.26%) |  |
| HISPANIC/LATINO | 12(0.62%) | 71(3.66%) |  |
| WHITE | 456(23.53%) | 1290(66.56%) |  |
| UNKNOWN | 106(5.47%) | 230(11.87%) |  |
| OTHER | 34(1.75%) | 106(5.47%) |  |
| **First_careunit (%)** |  |  | < 0.001 |
| CVICU | 159 (13) | 108 (16) |  |
| CCU | 53 (4) | 54 (8) |  |
| MICU | 388 (31) | 229 (34) |  |
| MICU/SICU | 418 (33) | 161 (24) |  |
| Neuro Intermediate | 21 (2) | 14 (2) |  |
| Neuro Stepdown | 12 (1) | 4 (1) |  |
| Neuro SICU | 18 (1) | 10 (1) |  |
| SICU | 112 (9) | 58 (9) |  |
| Trauma SICU TSICU | 77 (6) | 42 (6) |  |
| **Comorbidity (%)** |  |  |  |
| Myocardial infarction | 152 (12) | 115 (17) | 0.004 |
| Congestive heart failure | 238 (19) | 200 (29) | < 0.001 |
| Peripheral vascular disease | 98 (8) | 66 (10) | 0.174 |
| Cerebrovascular disease | 127 (10) | 93 (14) | 0.022 |
| COPD | 287 (23) | 191 (28) | 0.012 |
| Cirrhosis without [hypersplenism](javascript:;) | 77 (6) | 58 (9) | 0.058 |
| Diabetes | 320 (25) | 178 (26) | 0.763 |
| **Infection sites (%)** |  |  |  |
| Lower respiratory infection | 318 (25) | 210 (31) | 0.01 |
| Genitourinary tract infection | 260 (21) | 113 (17) | 0.036 |
| Intra abdominal infection | 38 (3) | 29 (4) | 0.193 |
| Bacteremia | 43 (3) | 22 (3) | 0.935 |
| Skin and skin structure infection | 61 (5) | 38 (6) | 0.55 |
| Musculoskeletal infection | 11 (1) | 7 (1) | 0.927 |
| Biliary tract infection | 10 (1) | 3 (0) | 0.561 |
| Systemic fungal infection | 61 (5) | 37 (5) | 0.646 |
| Other infection | 603 (48) | 320 (47) | 0.749 |
| **Laboratory tests^a^** |  |  |  |
| Platelet_mean (K/uL) | 167 (120, 234) | 166.5 (118.92, 237.5) | 0.793 |
| Lymphocytes_mean (K/uL) | 27.84 (1.2, 104.02) | 34.43 (1.25, 101.31) | 0.802 |
| Neutrophils_mean (K/uL) | 254.08 (10.05, 945.38) | 389.4 (10.46, 1070.51) | 0.046 |
| N/LP | 5.16 (2.72, 10.25) | 5.41 (3.16, 10.85) | 0.027 |
| N/LP level, n (%) |  |  | 0.006 |
| Low | 344 (27) | 141 (21) |  |
| Middle | 608 (48) | 360 (53) |  |
| High | 306 (24) | 179 (26) |  |
| WBC_max (K/uL) | 13.5 (9.3, 18.6) | 14.5 (10.6, 19.42) | 0.004 |
| Aniongap_max | 16 (13, 19) | 16 (14, 19) | 0.007 |
| Bicarbonate_min (mEq/L) | 21 (18, 23) | 21 (18, 23) | 0.321 |
| Bun_max (mg/dL) | 20 (14, 35) | 23 (15, 37) | 0.006 |
| Chloride_max (mEq/L) | 107 (103.25, 111) | 107 (103, 111) | 0.785 |
| Creatinine_max (μmol/L) | 1.1 (0.8, 1.6) | 1.1 (0.8, 1.5) | 0.288 |
| Glucose_max (mg/dl) | 137 (112.25, 181) | 149.5 (121, 208) | < 0.001 |
| Sodium_max (mEq/L) | 140 (137, 142) | 140 (137, 143) | 0.01 |
| Potassium_max (K/uL) | 4.3 (4, 4.8) | 4.5 (4.1, 5) | < 0.001 |
| **Severity scoring** |  |  |  |
| SAPS II | 34 (26, 42) | 37 (31, 46) | < 0.001 |
| SOFA_exclude platelet | 4 (3, 6) | 6 (4, 8) | < 0.001 |
| Charlson comorbidity index | 5 (3, 7) | 5 (4, 8) | 0.017 |
| **Treatments** |  |  |  |
| Vasoactive drug (%) | 482 (38) | 382 (56) | < 0.001 |
| Invasive ventilation (%) | 294 (23) | 372 (55) | < 0.001 |
| CRRT (%) | 1 (0) | 6 (1) | 0.009 |
| **Endpoints** |  |  |  |
| Length of ICU stay (day) | 1.91 (1.46, 2.82) | 4.21 (2.64, 7.51) | < 0.001 |
| Mortality_ICU (%) | 21 (2) | 75 (11) | < 0.001 |
| Mortality_ICU7 (%) | 29 (2) | 48 (7) | < 0.001 |
| Mortality_ICU28 (%) | 55 (4) | 93 (14) | < 0.001 |

Categorical data were presented as frequency (percentage), parametric continuous data were presented as median (interquartile ranges), whereas non-parametric continuous data were presented as median (interquartile ranges);

^a^Laboratory tests were calculated during the first 24 h since ICU admission of each included patients;

CVICU: Cardiac Vascular Intensive Care Unit; CCU: Coronary Care Unit; MICU: Medical Intensive Care Unit; MICU/SICU: Medical/Surgical Intensive Care Unit; SICU: Surgical Intensive Care Unit; COPD: Chronic Obstructive Pulmoriary Disease; SOFA: Sequential Organ Failure Assessment; SAPS II: Simplified acute physiology score II; AKI: Acute kidney injury; CRRT: continuous renal replacement therapy; ICU: intensive care unit.
