# Supplementary material for: Influence of the Initial Neutrophils to Lymphocytes and Platelets Ratio on the Incidence and Severity of Sepsis-Associated Acute Kidney Injury: A Double Robust Estimation Based on a Large Public Database
Source: Front Immunol. 2022 Jul 12;13:925494. doi: 10.3389/fimmu.2022.925494 (PMC9320191; doi:10.3389/fimmu.2022.925494)
Supplement: Supplementary file 14 [file Table_3.docx]

Supplementary Table 3 The results of Cox proportional hazards models and competing risk analyses on the basis of urinary output criteria

| Level |  | Model 1 | |  | Model 2 | |  | Model3 | |
| --- | --- | --- | --- | --- | --- | --- | --- | --- | --- |
|  |  | HR(95% CI) | P |  | HR(95% CI) | P |  | HR(95% CI) | P |
| **Cox proportional hazard models** |  |  |  |  |  |  |  |  |  |
| Low N/LP group |  | Ref | 1 |  | Ref | 1 |  | Ref | 1 |
| Middle N/LP group |  | 1.37(1.11, 1.69) | 0.003 |  | 1.31(1.06, 1.62) | 0.011 |  | 1.31(1.07, 1.62) | 0.010 |
| High N/LP group |  | 1.35(1.06, 1.71) | 0.013 |  | 1.29(1.01, 1.65) | 0.037 |  | 1.30(1.02, 1.64) | 0.033 |
| **Competing risk analyses** |  |  |  |  |  |  |  |  |  |
| Low N/LP group |  | Ref | 1 |  | Ref | 1 |  | Ref | 1 |
| Middle N/LP group |  | 1.36(1.11, 1.68) | 0.003 |  | 1.31(1.07, 1.61) | 0.010 |  | 1.31(1.06, 1.61) | 0.012 |
| High N/LP group |  | 1.34(1.06, 1.70) | 0.014 |  | 1.29(1.01, 1.63) | 0.039 |  | 1.28(1.01, 1.62) | 0.039 |

Model 1: univariate analysis; Model 2: adjusted for age, gender, initial SAPS II, SOFA scores excluding coagulation system, Charlson comorbidity index,

serum AG, serum bicarbonate, glucose, serum potassium, serum sodium, serum chloride, BUN, SCr, the use of vasoactive medication, CRRT, and invasive-MV;

Model 3: adjusted for age, serum chloride, SCr, SOFA scores excluding coagulation system, vasoactive medication, CRRT, and invasive-MV;

SOFA: Sequential Organ Failure Assessment; SAPS II: Simplified acute physiology II; AKI: Acute kidney injury; CRRT: Continuous Renal Replacement Therapy;

MV: machine ventilation; SCr: Serum Creatinine; N/LP: Neutrophil-to-Lymphocyte Platelet.
